# Novel Tdp1 Inhibitors Based on Adamantane Connected with Monoterpene Moieties via Heterocyclic Fragments

**DOI:** 10.3390/molecules26113128

**Published:** 2021-05-24

**Authors:** Aldar A. Munkuev, Evgenii S. Mozhaitsev, Arina A. Chepanova, Evgeniy V. Suslov, Dina V. Korchagina, Olga D. Zakharova, Ekaterina S. Ilina, Nadezhda S. Dyrkheeva, Alexandra L. Zakharenko, Jóhannes Reynisson, Konstantin P. Volcho, Nariman F. Salakhutdinov, Olga I. Lavrik

**Affiliations:** 1 N. N. Vorozhtsov Novosibirsk Institute of Organic Chemistry, Siberian Branch of the Russian Academy of Sciences, 9, Akademika Lavrentieva Ave., 630090 Novosibirsk, Russia; amunkuev@nioch.nsc.ru (A.A.M.); mozh@nioch.nsc.ru (E.S.M.); suslov@nioch.nsc.ru (E.V.S.); korchaga@nioch.nsc.ru (D.V.K.); volcho@nioch.nsc.ru (K.P.V.); anvar@nioch.nsc.ru (N.F.S.); 2 Institute of Chemical Biology and Fundamental Medicine, Siberian Branch of Russian Academy of Sciences, 8, Lavrentiev Ave., 630090 Novosibirsk, Russia; arinachepanova@mail.ru (A.A.C.); garonna3@mail.ru (O.D.Z.); katya.plekhanova@gmail.com (E.S.I.); elpida80@mail.ru (N.S.D.); a.zakharenko73@gmail.com (A.L.Z.); lavrik@niboch.nsc.ru (O.I.L.); 3 School of Pharmacy and Bioengineering, Keele University, Hornbeam Building, Staffordshire ST5 5BG, UK

**Keywords:** topoisomerase 1, monoterpenoid, cancer, DNA repair enzyme, SAR, molecular modeling, chemical space

## Abstract

Tyrosyl-DNA phosphodiesterase 1 (Tdp1) is a promising target for anticancer therapy due to its ability to counter the effects topoisomerase 1 (Top1) poison, such as topotecan, thus, decreasing their efficacy. Compounds containing adamantane and monoterpenoid residues connected via 1,2,4-triazole or 1,3,4-thiadiazole linkers were synthesized and tested against Tdp1. All the derivatives exhibited inhibition at low micromolar or nanomolar concentrations with the most potent inhibitors having IC_50_ values in the 0.35–0.57 µM range. The cytotoxicity was determined in the HeLa, HCT-116 and SW837 cancer cell lines; moderate CC_50_ (µM) values were seen from the mid-teens to no effect at 100 µM. Furthermore, citral derivative **20c**, α-pinene-derived compounds **20f**, **20g** and **25c,** and the citronellic acid derivative **25b** were found to have a sensitizing effect in conjunction with topotecan in the HeLa cervical cancer and colon adenocarcinoma HCT-116 cell lines. The ligands are predicted to bind in the catalytic pocket of Tdp1 and have favorable physicochemical properties for further development as a potential adjunct therapy with Top1 poisons.

## 1. Introduction

Cancer is one of the leading causes of death worldwide; according to the International Agency for Research on Cancer (IARC), in 2018 new cancer cases were estimated to be approximately 18.1 million, with the number of fatal outcomes being almost 10 million worldwide [1].

The camptothecin derivatives irinotecan and topotecan are frontline cancer drugs used for the treatment of breast, small-cell lung, lymphomas, cervical, colorectal and ovarian cancers [2,3,4,5]. Their target is Topoisomerase 1 (Top1), an enzyme that catalyzes the process of relieving the torsional DNA strain during replication, transcription and chromatin remodeling [6] by generating a reversible single-stranded break and covalently attaching to the 3′-end, followed by religation and releasing of DNA. The camptothecins, or Top1 poisons, stabilize the Top1−DNA complex [7,8], thereby inhibiting the religation process that eventually leads to cell death [6]. A major disadvantage of Top1 poisons-based therapy is the ability of the cancer cells’ DNA repair systems to remove the lesions, preventing cell death. One of the DNA repair enzymes is tyrosyl-DNA-phosphodiesterase 1 (Tdp1), which is capable of cleaving different adducts from the DNA 3′-end restoring its integrity [9]. On the one hand, Tdp1 is overexpressed in some types of cancer, such as non-small lung cancer [10] and glioblastoma [11]. On the other hand, it has been shown that Tdp1 knockout mice and Tdp1-deficient cells are hypersensitive to Top1 inhibitors [12,13,14]. Therefore, the inhibition of Tdp1 could increase the efficacy of Top1 poisons, making it a promising anticancer target [15].

**Figure 1:**
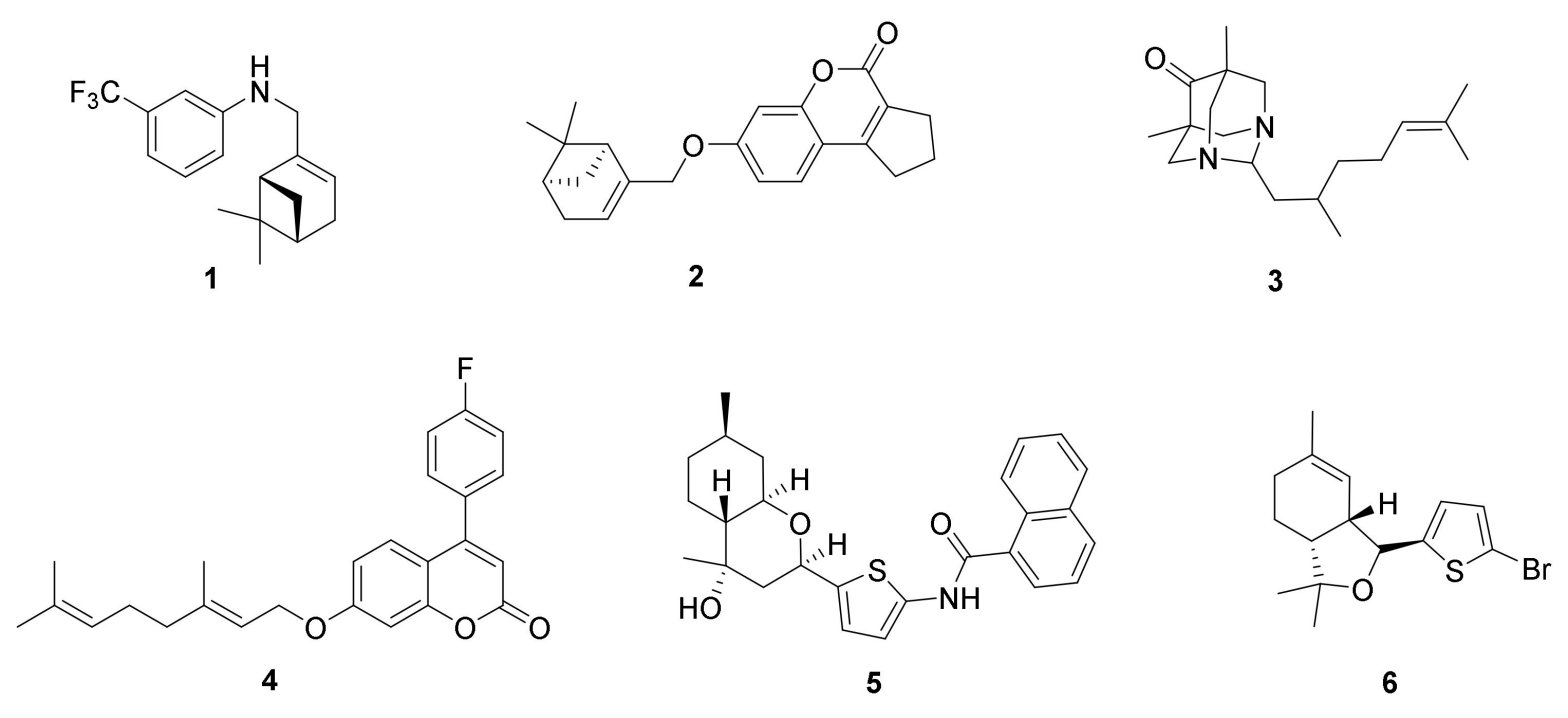
The molecular structures of known monoterpenoid-derived Tdp1 inhibitors.

Monoterpenoid is a useful moiety in potent Tdp1 inhibitors. e.g., the aniline derivative **1** with a myrtenal moiety has inhibitory activity at submicromolar concentrations (Figure 1) [16]; coumarin-based (-)-myrtenal derivative **2** potentiated the cytotoxicity of camptothecin in cancer cells [17]; it has also been shown that **3,** comprising diazaadamantane and citronellal residues, inhibits Tdp1 with IC_50_ (half maximal inhibitory concentration) values of ~15 µM [18]; geranyl derivative **4** enhances the topotecan antitumor effect in in vitro and in vivo tumor models [19]; octahydro-2*H*-chromene-derived compound **5** was found to be an effective Tdp1 inhibitor with an IC_50_ value of 2 µM [20]. Finally, isobenzofuran **6**, containing a thiophene fragment, has a synergistic effect with topotecan in wild type cells, but not in Tdp1 knockout cells [21].

Adamantane derivatives enact many types of biological activity, including antiviral, actoprotective, antituberculosis and cannabimimetic properties, with some in clinical use [22]. Furthermore, the adamantane moiety is associated with the enhanced lipophilicity and stability of the drugs [22].

**Figure 2:**
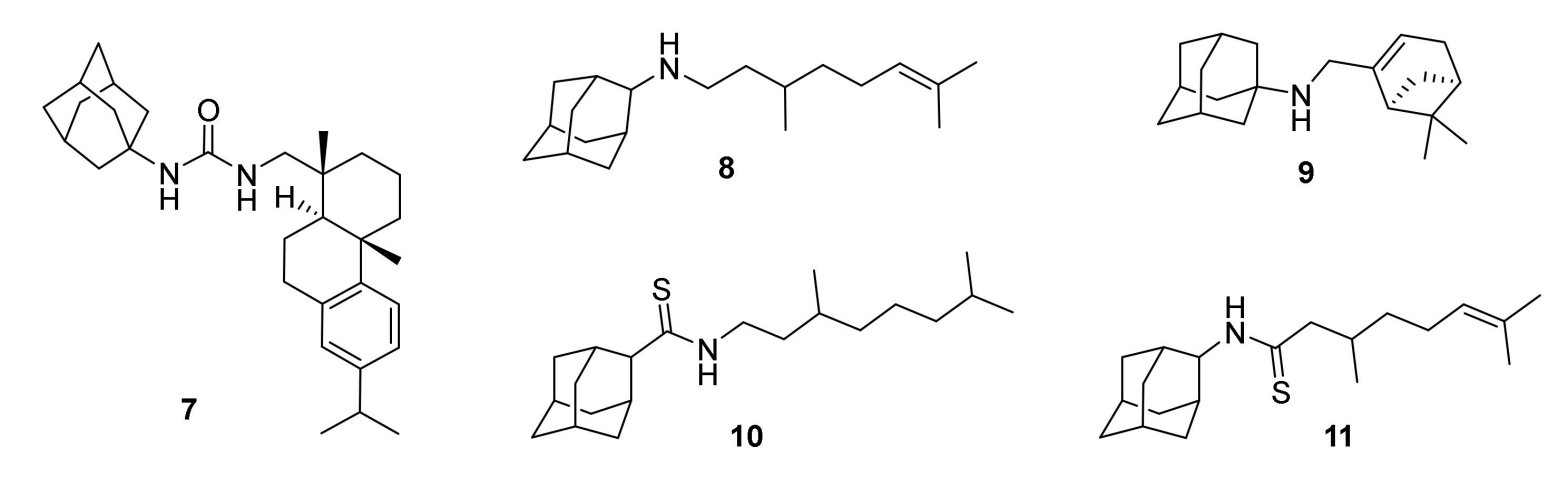
The molecular structures of Tdp1 inhibitors containing adamantane and terpene moieties.

Terpenoids linked to an adamantane moiety were also found to exhibit Tdp1 activity; the dehydroabietyl amine derivative with a 1-adamantyl fragment **7** (Figure 2) not only had inhibitory activity against Tdp1, but also enhanced the antitumor effect of temozolomid in glioblastoma cells [23]; adamantyl-derived compounds containing citronellyl **8** and (+)-myrtenyl **9** fragments inhibited Tdp1 activity in the low micromolar range and citronellol derivative **8** potentiated the cytotoxicity of topotecan in the human colorectal carcinoma cell line HCT-116 [24]; moderate activity against Tdp1 was found for thioamides having adamantane and 3,7-dimethyloctyl **10** and citronellyl **11** moieties, with compound **10** showing synergetic effect with topotecan [25].

1,2,4-Triazole derivatives are broadly used as antifungal, antimigraine and antiviral agents (see Figure S1 in the Supplementary Materials). Additionally, drugs with a 1,3,4-thiadiazole fragment are used mainly as antibacterial agents, such as acetazolamide, a carbonic anhydrase inhibitor, which is used for the treatment of glaucoma [26] and high-altitude illness [27].

**Figure 3:**
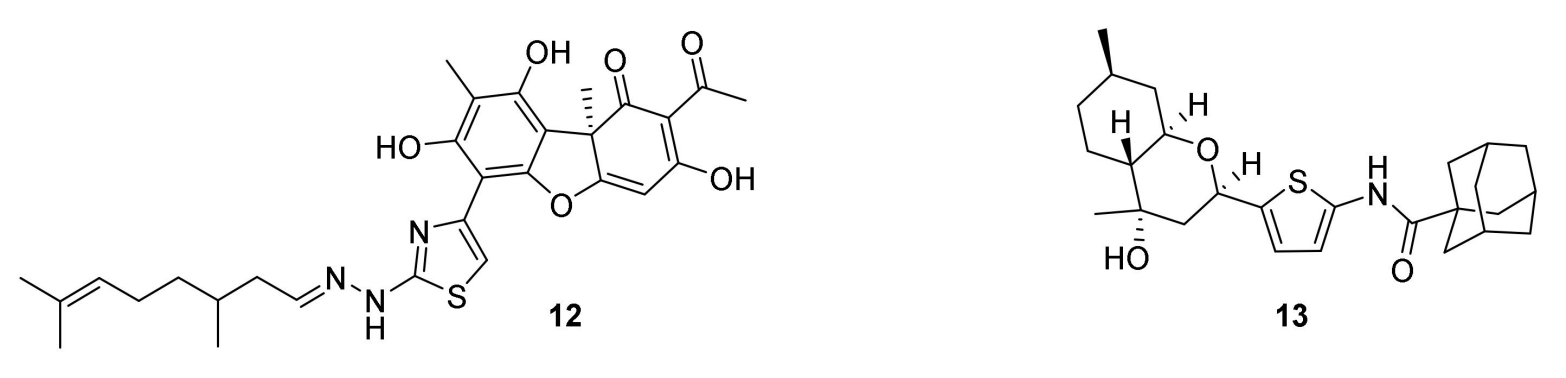
The molecular structures of Tdp1 inhibitors with a heterocyclic linker.

There are few examples of Tdp1 inhibitors having a five-membered heterocyclic core. The 1,3-thiazole moiety is as a linker between a geranyl fragment and usnic acid moiety in compound **12** (Figure 3), with the hybrid molecule demonstrating synergetic effects in combination with topotecan [28]. Another example of using a heterocyclic core as a linker is compound **13**, where a thiophene residue connects an octahydro-2*H*-chromen-4-ol scaffold with an adamantyl residue with an IC_50_ value of 1.2 µM [29].

In this study, we aimed at synthesizing several conjugates linking adamantane and monoterpenoid residues via 1,2,4-triazole and 1,3,4-thiadiazole linkers, to derive their structure–activity relationship for Tdp1 activity. This study is a continuation of our previous work on adamantyl conjugates with monoterpenoid residues capable of inhibiting Tdp1 to expand and improve the chemical matter of potent Tdp1 inhibitors.

## 2. Results and Discussion

### 2.1. Chemistry

**Scheme 1:**
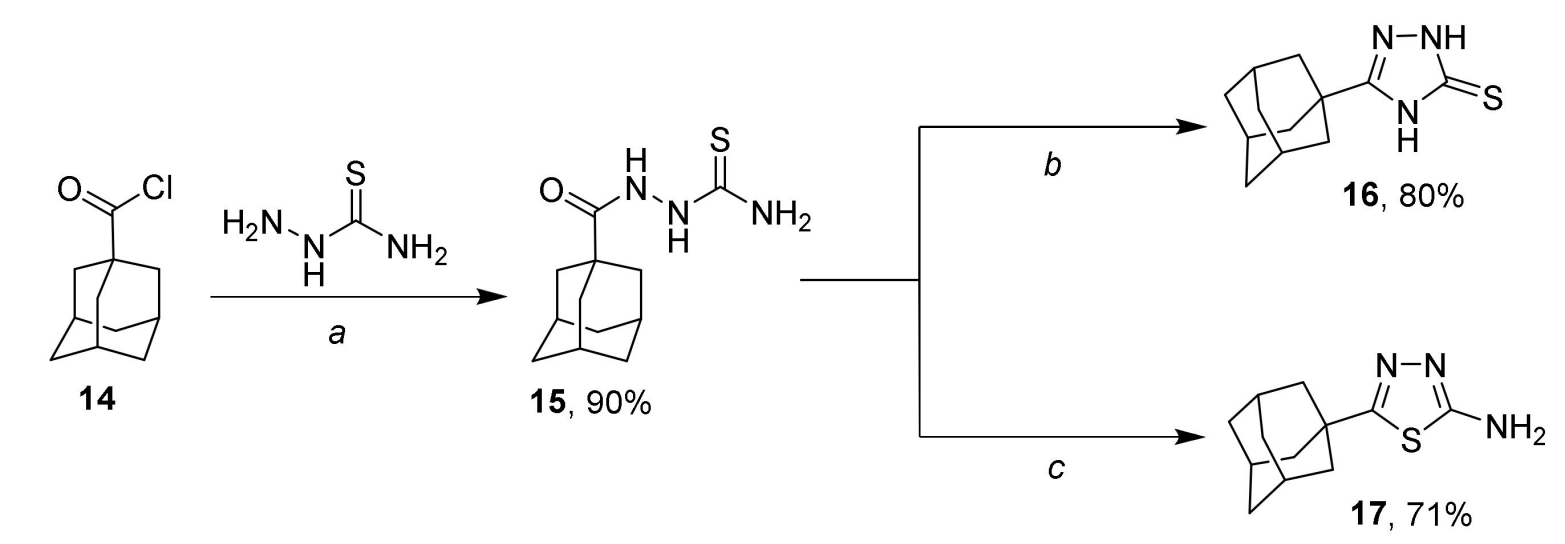
Synthesis of starting heterocyclic compounds **16** and **17**. Reagents and conditions: (**a**) THF, 0 °C, then r.t., (**b**) NaOH, H_2_O, Δ, (**c**) H_2_SO_4_ conc., r.t.

According to Scheme 1, we synthesized starting compound **15** by reacting commercially available adamantane-1-carbonyl chloride **14** with thiosemicarbazide, which was used in a double excess for capturing HCl. This method allows the synthesis of the desired compound at an excellent yield. Synthesis of 1,2,4-triazole **16** was performed by the cyclocondensation of compound **15** in an aqueous solution of sodium hydroxide under refluxing conditions with 80% yield, as described previously [30]. 2-Amino-1,3,4-thiadiazole **17** was synthesized with a 71% yield after recrystallization from EtOH following a procedure previously described [31], which involved the treatment of starting compound **15** with concentrated sulfuric acid at room temperature.

**Scheme 2:**
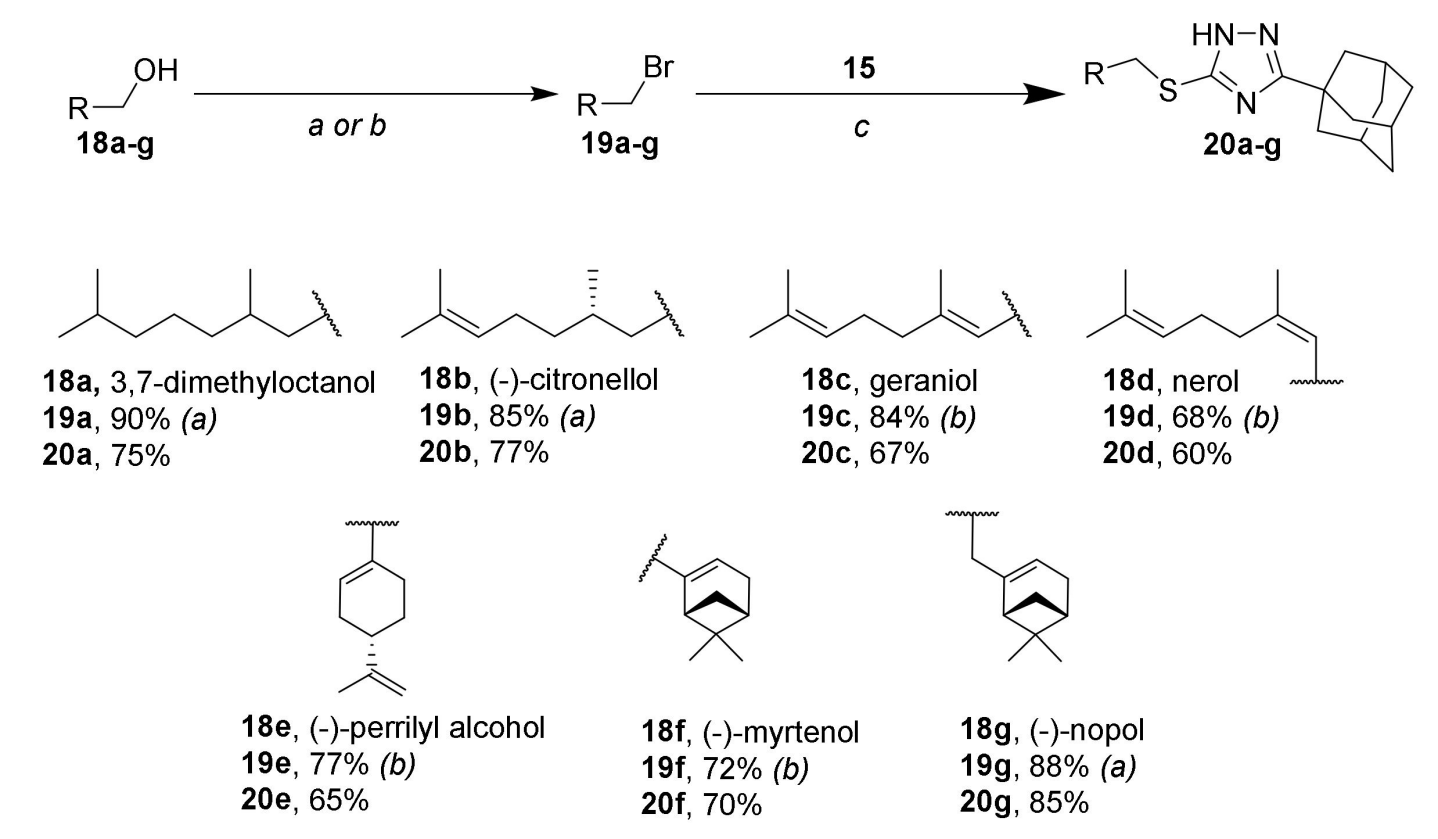
Synthesis of bromides **19a**–**g** and target 1,2,4-triazole derivatives **20a**–**g**. Reagents and conditions: (**a**) NBS, PPh_3_, CH_2_Cl_2_, r.t., (**b**) PBr_3_, 0 °C, (Et_2_O for **18b**, **18e**, **18f** and THF for **18d**), (**c**) MeONa, MeOH, 60 °C.

For the attachment of monoterpenoid residues to compound **16**, we obtained bromides **19a**–**g** using the previously reported methods [32,33,34]. The treatment of alcohols **18c**–**f** with PBr_3_ in Et_2_O or THF resulted in the bromides **19c**–**f**. The reaction of monoterpene alcohols **18a**, **18b** and **18g** with NBS and PPh_3_ resulted in compounds **19a**, **19b** and **19g** (Scheme 2).

The further alkylation of compound **16** with bromides in the presence of MeONa in MeOH led to the formation of the desired adamantane derivatives of 1,2,4-triazole bearing monoterpenoid fragments **20a**–**g**. All the reactions proceeded smoothly with moderate to very good yields. Interestingly, the interaction of **15** with geranyl bromide **19c,** with the formation of **20c,** led to its (*Z*)-isomer **20d** as an admixture with a ratio of 9:1 (**20c**:**20d**), as assessed by NMR. A similar situation was observed for the reaction of **15** with bromide **19d**; in this case the ratio between **20d** and **20c** was 9.5:0.5 (Scheme 2). This ratio can be explained by a partial isomerization process of bromides or target products as described previously [35].

**Scheme 3:**
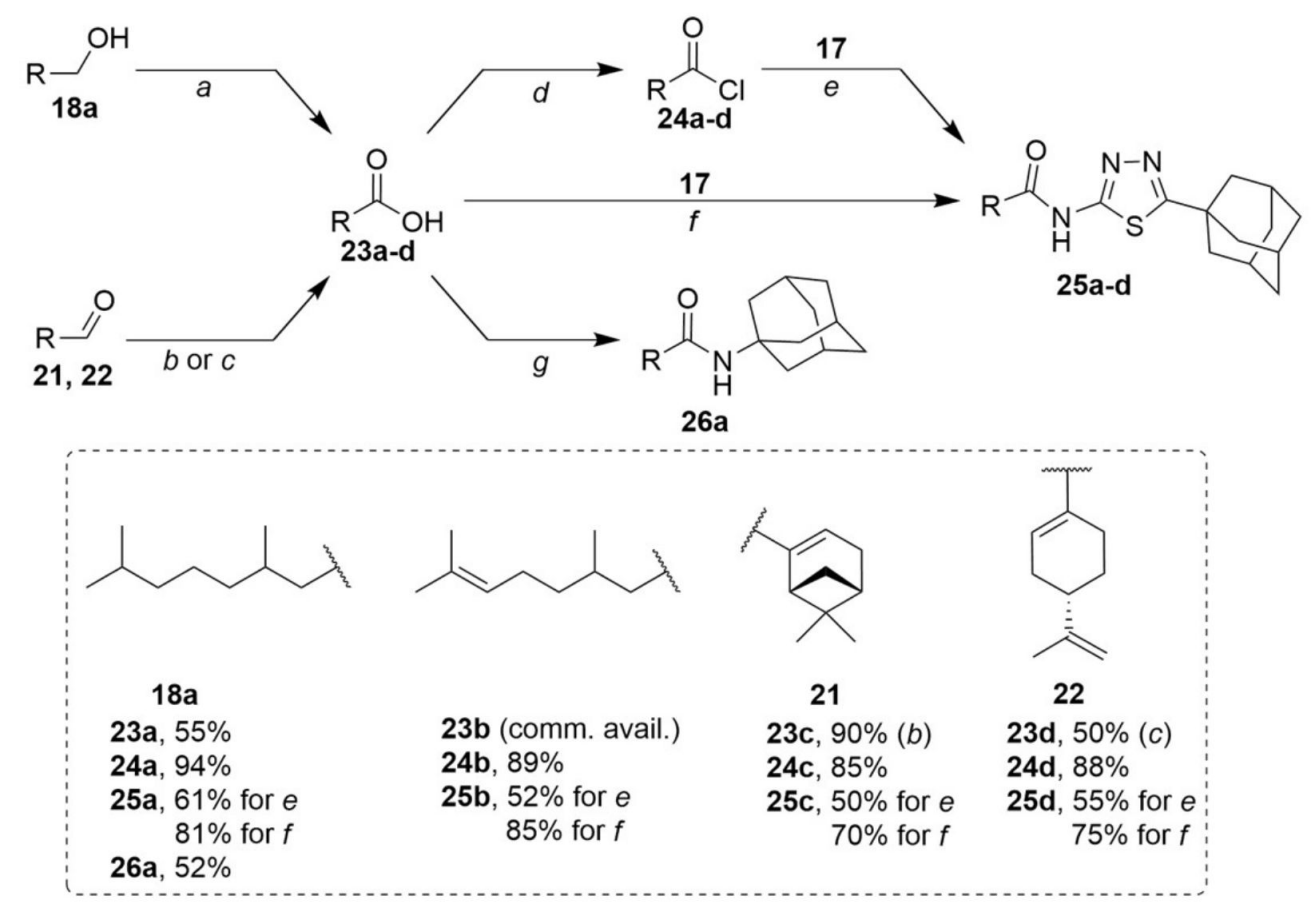
Synthesis of desired amides **25a**–**d** and **26a**. Reagents and conditions: (**a**) CrO_3_, H_2_SO_4_, acetone, H_2_O; (**b**) NaClO_2_, KH_2_PO_4_, DMSO, H_2_O, r.t.; (**c**) NaClO_2_, KH_2_PO_4_, H_2_O_2_, CH_3_CN, H_2_O, r.t.; (**d**) SOCl_2_, benzene, Δ; (**e**) toluene, Et_3_N, r.t.; (**f**) T3P, Py, EtOAc (r.t. for **25a,b**, reflux for **25c,d**); (**g**) 1-aminoadamantane, T3P, Py, EtOAc, r.t.

In order to modify amine **17**, carboxylic acids **23a**,**c**,**d** were obtained b**y** applying previously reported procedures [36,37,38]. 3,7-Dimethyloctan-1-ol **18a** was oxidized by Jones reagent to obtain carboxylic acid **23a** [36]. Acids **23c** and **23d** were synthesized using Pinnick oxidation [37,38], which involved the treatment of aldehydes **21** and **22** with sodium chlorite at mild acidic conditions. The yields of compounds **23c** and **23d** were 85% and 50%, respectively (Scheme 3).

One of the most common methods to activate a carboxylic acid functional group is converting it to the corresponding carbonyl chloride. Utilizing this protocol, we synthesized acid chlorides **24a**–**d** from acids **23a**, **23c** and **23d** and commercially available citronellic acid **23b** using SOCl_2_, which were all used in further reactions without purification. The reaction of acid chlorides with amine **17** resulted in 1,3,4-thiadiazole derivatives **25a**–**d** containing 1-adamantyl moiety and monoterpenoid residues at acceptable yields.

Propylphosphonic acid anhydride T3P is a useful coupling reagent that generates water-soluble by-products easily separated from reaction mixtures [39]. Applying T3P allowed us to synthesize amides **25a**–**d** at good yields and with high purity. Full conversion was observed after 24 h at room temperature. α,β-Unsaturated acids **23c** and **23d** were found to react too slowly with amine **17** at room temperature. Carrying out the reaction under refluxing conditions for 24 h in EtOAc followed by silica gel column chromatography gave compounds **25c** and **25d** in very good yields (Scheme 3). Compound **26a** was obtained at a good yield using the same procedure. The rest of the amides containing citronellic and (-)-myrtenic acid moieties were synthesized as previously reported [40].

Thus, starting from compound **15**, we synthesized a series of conjugates, with 1,2,4-triazole and 1,3,4-thiadiazole as linkers. 1,2,4-Triazol-2-thione **16** was modified by alkylation with the corresponding bromides in good yields. To obtain 1,3,4-thiadiazole amides **25a**–**d**, two methods were utilized: (1) converting carboxylic acids into corresponding acid chlorides followed by the acylation of amine **17**; (2) direct interaction of carboxylic acids with 1,3,4-thiadiazole **17** in the presence of coupling reagent T3P. The second approach proved to be more convenient due to the expedient work-up of the reaction mixtures, the high target compound yields, and the high purity of the amides.

### 2.2. Biological Assays

In order to assess the inhibitory activity of the synthesized compounds, a fluorophore quencher-coupled DNA-biosensor for the real-time measurement of Tdp1 cleavage activity was used [41]. Furamidine was used as a positive control, which is a commercially available Tdp1 inhibitor (IC_50_ 1.23 µM) [42], and the results are presented in Table 1. **25d** could not be measured due to its poor solubility.

According to Table 1, of the triazoles **20a**–**d** with aliphatic chains, the saturated 3,7-dimethyloctane derivative **20a** was the most active as compared to its unsaturated counterparts. The thio-derivatives **20c** and **20d**, cis-trans isomers, showed practically the same IC_50_ values (~5 μM). Interestingly, the bicyclic derivative **20f** had an IC_50_ value of 7.50 μM, whilst **20g** substituted with a (-)-nopol moiety was in the submicromolar range (0.57 μM). The same pattern of saturated and unsaturated derivatives could be observed for the amides **25a** and **25b** as for the triazoles. Additionally, **25c** with a (-)-myrtenic moiety had activity comparable to **20g,** but much better activity than **20f**, its triazole counterpart.

**Scheme 4:**
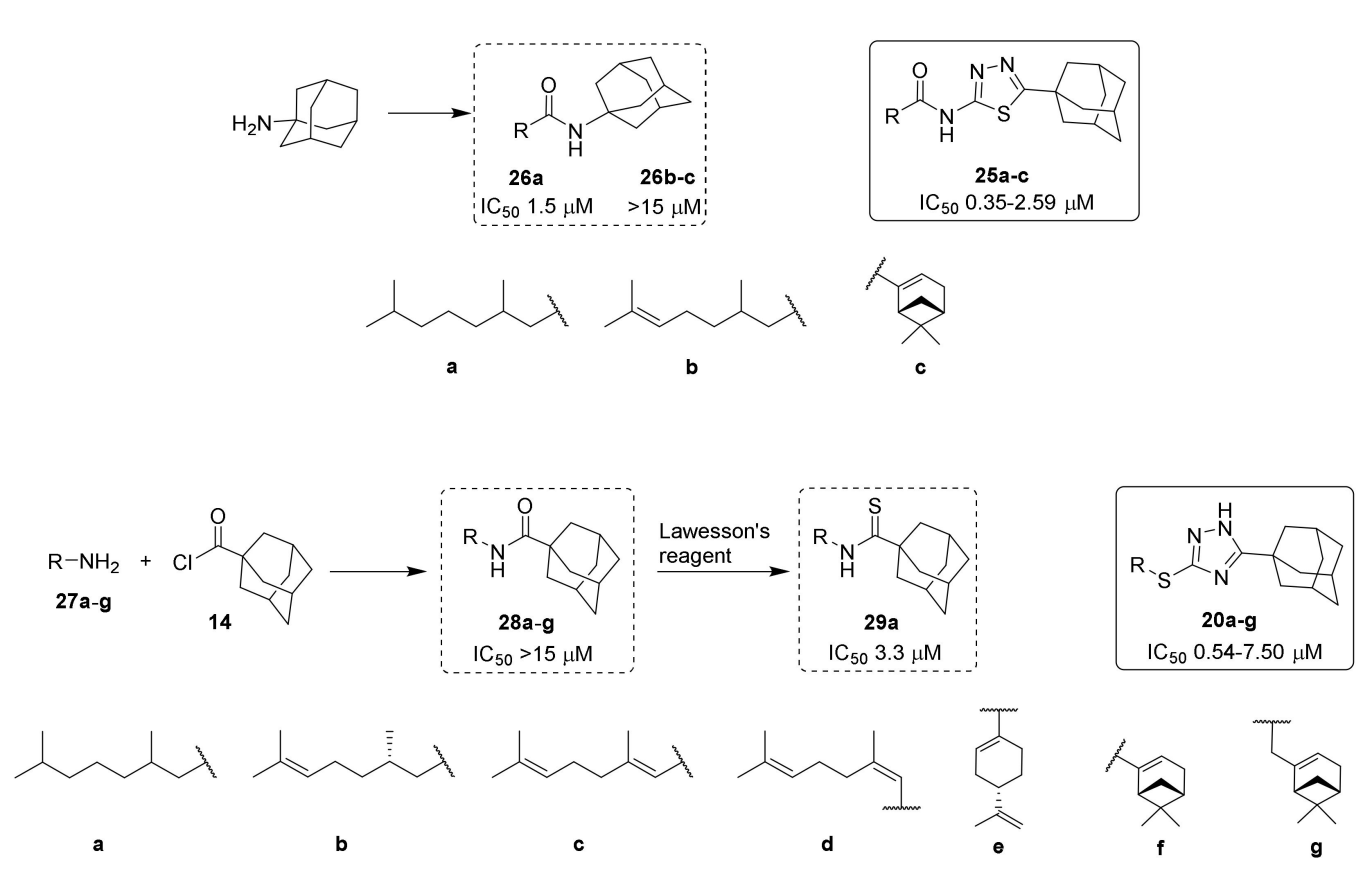
The synthesis of amides **26b**, **28a**, **28d**, and **29a** was previously described [25]. Compounds **26c**, **28b**, **28c**, and **28e**–**g** were reported previously [40].

The 1,3,4-thiadiazole ring plays an important role in the inhibition of Tdp1, i.e., the amide derivatives **26** (see Scheme 4), composed of a monoterpenoid and 1-aminoadamantane [25,40], were less active, whereas their structural analogues, the thiadiazoles (**25**), showed good activity. Furthermore, by comparing **20a**–**g** with the previously reported amides **28a**–**g** [40] and thioamide **29a** [25] (Scheme 4), also made up by 1-adamantane and monoterpenoid fragments, we see that the replacement of the amide or thioamide functional groups with a 1,2,4-triazole linker increased the potency for Tdp1.

The cytotoxicity of compounds was investigated using HeLa (cervical cancer), SW837 and HCT-116 (colon cancers) cell lines, and the results are shown in Table 1 and Figure S2 in the Supplementary Materials. The 1,3,4-thiadiazole derivatives (**25**) had a minor effect on the viability of the cell lines, with only two measured CC_50_ values under 100 µM, whereas the triazole series (**20**) had a considerable impact, with CC_50_ values as low as 15 µM. In general, the HeLa cell line was less sensitive to the inhibitors than the HCT-116 and SW837 cells. Leading compounds **20c** and **20g** were also tested on the noncancerous cell line HEK293A; they had no effect on cell viability (data not shown) at concentrations up to 30 μM.

**Figure 4:**
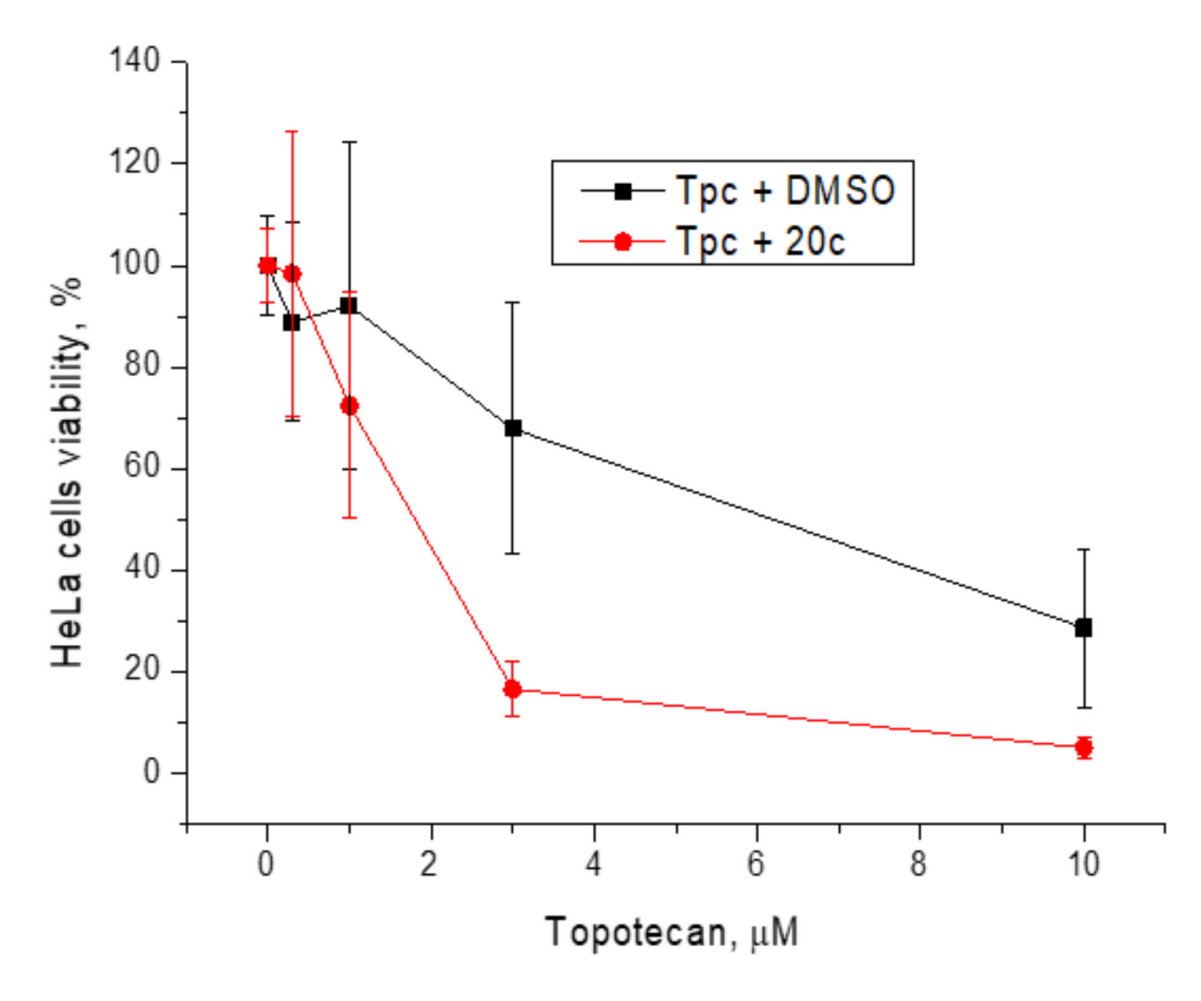
Sensitizing effect of **20c** at 5 μM non-toxic concentration on the viability of HeLa cells.

The potential for sensitizing the cancer cell lines to topotecan using the Tdp1 inhibitors was investigated using the MTT assay. Neutralizing the enzymatic activity of Tdp1 by blocking access to its catalytic site using a small molecule should sensitize the cytotoxic potential of topotecan, since the complex of Top1 with DNA cannot be unraveled. Interestingly, other repair mechanisms than Tdp1 may also be at play in the cancer cells mitigating the cytotoxicity of topotecan. Nontoxic concentrations of the adamantane derivatives (5 μM) were used for the HeLa cell line; the dose response for topotecan was measured and the corresponding CC_50_ values were derived, and the results are shown in Figure 4 for **20c** and in Table 2 for all the derivatives.

For HeLa cells, increased cytotoxicity was seen when the adamantanes and topotecan were combined. The most effective sensitizer was **20c**, followed by all the derivatives containing bicyclic fragments. Compound **25b**, with an acyclic substituent, exhibited a moderate sensitizing effect, but due to its poor solubility it was not considered further. The combination index (CI) for different concentrations of topotecan and **20c** at 5 μM was in the range 0.49–0.82, and at 10 μM of **20c,** it was 0.23–0.78. This indicates a synergistic effect of topotecan and **20c**. The CI values were calculated with the CompuSyn version 1.0 software. For the HCT-116 cells, the synergistic effects of the compounds were different than for the HeLa cells. The most potent compound was **20g**, followed by **20c**, **20e**, **25a** and **25c**. **20b** and **25b** also showed moderate sensitization. The CI plot at a different fraction affected (Fa) was determined for topotecan (5, 15, 150, 500 and 1500 nM) and **20g** (1, 3, 10, 20 μM). The CI values for most of the combinations correspond to a synergistic effect (CI < 1, see Figure S3 in the Supplementary Materials).

### 2.3. Molecular Modeling

**Figure 5:**
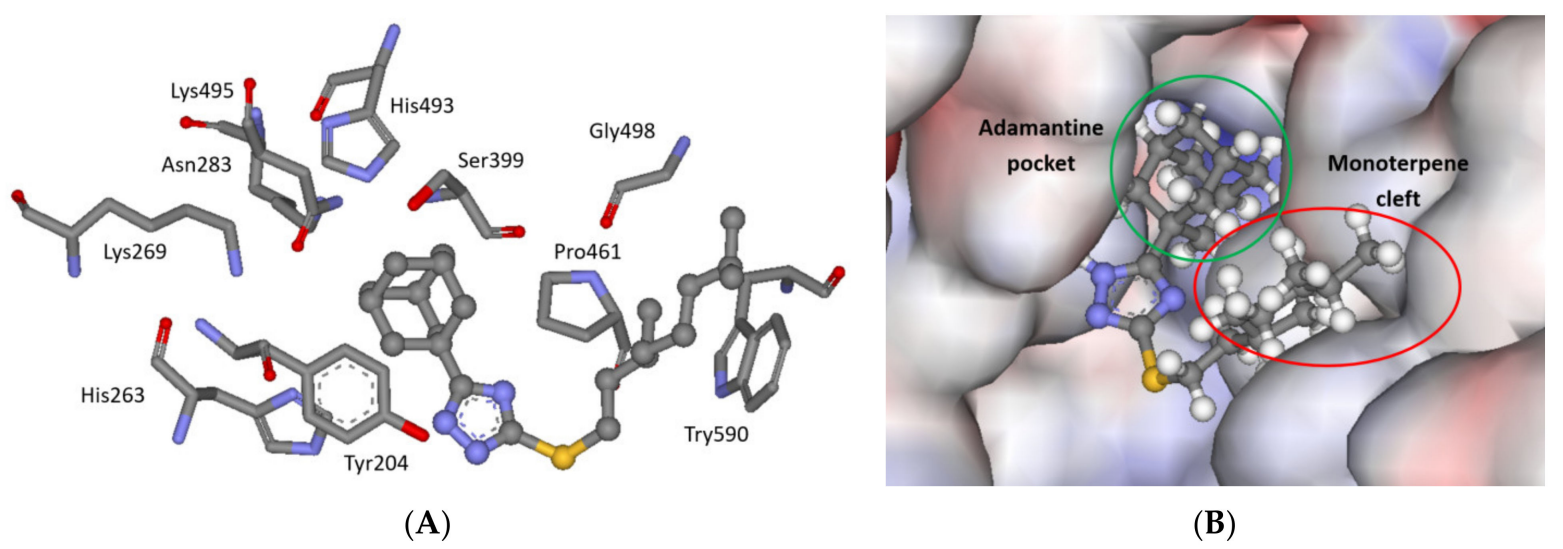
The docked pose of **20a** in the binding site of Tdp1 as predicted by the ChemPLP scoring function. (**A**) The predicted configuration is shown in the ball-and-stick format and the adjacent amino acid residues are depicted in the stick format; the hydrogen atoms are not shown for clarity. The amino acids forming the pocket accommodating the adamantine cage are Tyr204, His263, Lys269, Asn283, Ser399, His493 and Lys495; the lipophilic cleft occupied by the aliphatic monoterpene is made up by Pro461, Gly498 and Try590. (**B**) The protein surface is rendered; blue depicts regions with a partial positive charge on the surface; red depicts regions with a partial negative charge and grey shows neutral areas. The ligand occupies the catalytic binding pocket, blocking access to it.

Thirteen adamantane–monoterpene derivatives were docked into the binding site of the Tdp1 structure (PDB ID: 6DIE, resolution 1.78 Å) [43] with three water molecules retained (HOH814, 821 and 1078). It has been shown that keeping these crystalline water molecules improves the prediction quality of the docking scaffold (see the Methodology section for further information) [44]. The binding predictions of the four scoring functions used are given in Table S1 in the Supplementary Materials; all the ligands show reasonable scores, but no linear correlation was observed with the measured IC_50_ values. Nevertheless, when the scores of the ligands with IC_50_ values >15 µM were calculated, a clear statistical difference was seen between their average and that of the active compounds, with the exception of ChemScore (CS) (see Table S1). When the docked poses were analyzed, it emerged that the adamantane and monoterpene moieties were driving the binding, i.e., adamantane occupied the pocket containing both the catalytic histidine amino acid residues (His263 and 493) and the monoterpene fit into a lipophilic cleft. No hydrogen bonding interactions were predicted, indicating that the interaction was dominated by lipophilic contacts. The predicted configuration of **20a** is shown in Figure 5 as an example of the dominant binding mode.

A subset of ligands with varied affinity to Tdp1 is **20a** (0.54 µM), **20b** (1.5 µM) and **20c** (5.30 µM). These have similar structures; only the saturation state of the aliphatic chain is different, and thus an interesting trend is seen. The binding of a ligand to an enzyme is governed by Equation (1).

(1) $$\Delta G_{Bind}=\Delta G_{Dehydration}+\Delta G_{Configuration}+\Delta G_{Intramol.\;\;binding}$$

The three ligands were all predicted to bind to Tdp1 in the same manner, i.e., the configuration and intramolecular binding terms would be the same. When the log *p* values are considered, since log P correlates with Δ*G_Dehydration_* [45], an excellent negative correlation is seen (R^2^—0.894, see Figure S4A). It is therefore clear that the interaction of these ligands is governed by an entropic push from the water phase and lipophilic contacts with the binding pocket’s surface.

The same trend was seen when structurally near-identical ligands **20f** (7.50 µM) and **20g** (0.57 µM) were compared; they had the same predicted binding, but **20g** had a higher log *p* value than **20f** (6.1 vs. 5.8). This trend was repeated for the **20a** (0.54 µM) and **28a** (>15 µM) pair, with the same binding pose but substantially different log *p* values (6.6 vs. 5.1). For **20g** (0.57 µM) and **28g** (>15 µM), a structurally similar pair, the latter was not docked into the catalytic binding pocket, i.e., it did not fit into it and therefore did not fulfill the Δ*G_Configuration_* term in Equation (1). Interestingly, **28g** docked into the same site as our previously reported adamantine–monoterpene series [25]. Finally, the difference in the **25a** (0.45 µM) and **26a** (>15 µM) pair can be explained by the low molecular weight of the latter (383.6 vs. 299.5 g mol^−1^), as relatively small ligands have decreased binding affinity compared to their much bigger counterparts (see Chemical Space section and Figure S4B). This can be explained in terms of the smaller molecules having relatively few intramolecular interactions with the enzyme, leading to the third term in Equation (1) being small. In conclusion, the activity, or inactivity, of the ligands depends on their lipophilicity, as well as their fitting into the binding pocket (configuration) and finally having sufficient intramolecular interactions with the enzyme.

### 2.4. Chemical Space

The calculated molecular descriptors MW (molecular weight), log P (water–octanol partition coefficient), HD (hydrogen bond donors), HA (hydrogen bond acceptors), PSA (polar surface area) and RB (rotatable bonds) are given in Table S2 in the Supplementary Materials. Interestingly, when the molecular descriptor numbers were correlated with the IC_50_ values, MW showed good correlation with R^2^—0.5638, and HA (R^2^—0.2267) and PSA (R^2^—0.2242) also had reasonable correlations (see Figure S4 in the Supplementary Materials). A correlation between the molecular descriptor values and their corresponding binding efficacy to Tdp1 has been previously seen for deoxycholic acid derivatives, with MW having an R^2^ of 0.452, and 0.316 for RB [46]. The values of the molecular descriptors all lie within the lead- and drug-like chemical space, except for log P, which ranged from 4.7 to 6.6, thus reaching into the known drug space (for the definition of lead-like, drug-like and known drug space regions, see [47] and Table S3).

The known drug indexes (KDIs) for the ligands were calculated to gauge the balance of the molecular descriptors (MW, log P, HD, HA, PSA and RB). This method is based on the analysis of drugs in clinical use, i.e., the statistical distribution of each descriptor is fitted to a Gaussian function and normalized to 1, resulting in a weighted index. Both the summation of the indexes (KDI_2a_) and multiplication (KDI_2b_) methods were used [48], as shown for KDI_2a_ in Equation (2) and for KDI_2b_ in Equation (3); the numerical results are given in Table S2 in the Supplementary Materials.

KDI_2a_ = I_MW_ + I_log P_ + I_HD_ + I_HA_ + I_RB_ + I_PSA_ (2)

KDI_2b_ = I_MW_ × I_log P_ × I_HD_ × I_HA_ × I_RB_ × I_PSA_ (3)

The KDI_2a_ values for the ligands range from 4.82 to 5.59, with a theoretical maximum of 6 and an average of 4.08 (±1.27) for known drugs. These values are very good, since most of the descriptors lie within the lead- and drug-like boundaries of chemical space, except log P. The KDI_2b_ ranges from 0.23 to 0.64, with a theoretical maximum of 1 and with a KDS average of 0.18 (±0.20). Again, good values were obtained for the ligands even though the KDI_2b_ index is more sensitive than KDI_2a_ to outliers, since the multiplication of small numbers leads to smaller numbers. It can be concluded that the ligands are biocompatible as compared to drugs in clinical use.

## 3. Materials and Methods

### 3.1. Chemistry

All chemicals were purchased from commercial sources (Sigma Aldrich, Acros Organics) and used without further purification. ^1^H and ^13^C NMR spectra were recorded on a Bruker AV-300 spectrometer (300.13 MHz and 75.46 MHz, respectively), a Bruker AV-400 (400.13 MHz and 100.61 MHz), a Bruker DRX-500 (500.13 MHz and 125.76 MHz) and on a Bruker Advance—III 600 (600.30 MHz and 150.95 MHz). Residual chloroform signals were used as references (δ_H_ 7.24, δ_C_ 76.90 ppm). Compound structures were determined by analyzing the ^1^H-NMR spectra and ^1^H–^1^H 2D homonuclear correlation (COSY, NOESY), J-modulated ^13^C NMR spectra (JMOD), and ^13^C–^1^H 2D heteronuclear correlation with one-bond (HSQC, ^1^J = 145 Hz) and long-range spin–spin coupling constants (HMBC, ^2,3^J = 7 Hz). Mass spectra (70 eV) were recorded on a DFS Thermo Scientific high-resolution mass spectrometer. The conversion of starting materials was analyzed by thin-layer chromatography, which was performed on Merck plates (UV-254). A PolAAr 3005 polarimeter was used to measure optical rotations ([α]_D_). Target compounds were isolated by column chromatography (SiO_2_; 63–200 µm; *Macherey-Nagel*). Melting points were measured on a Mettler Toledo FP900 Thermosystem apparatus. Spectral and analytical measurements were carried out at the Multi-Access Chemical Service Center of Siberian Branch of Russian Academy of Sciences (SB RAS). The atom numeration of the substances is provided for the assignment of signals into the NMR spectra, and differs from that in IUPAC. The spectra are shown in the Supplementary Materials, Figures S5–S40.

Synthesis of 2-(adamantane-1-carbonyl)hydrazine-1-carbothioamide **15**

A solution of adamantane-1-carbonyl chloride (10.0 g, 50.4 mmol) in 30 mL of THF was added to a 0 °C suspension of thiosemicarbazide (10.08 g, 110.8 mmol) in 200 mL of THF while stirring. The resulting mixture was stirred at room temperature overnight followed by evaporating the solvent on a rotary evaporator. To the reaction mixture was added water; the solid mass was filtered off, washed with water thoroughly and dried. The yield of the compound **15** was 11.48 g (90%); the product was isolated as a white solid.

Synthesis of 5-(adamantan-1-yl)-2,4-dihydro-3*H*-1,2,4-triazole-3-thione **16**

A mixture containing 5.0 g (8.0 mmol) of 2-(adamantane-1-carbonyl) hydrazine-1-carbothioamide **15** and 1 M solution of NaOH (25 mL) was refluxed for 3 h. After cooling to room temperature, the mixture was neutralized using concentrated HCl. The precipitate was collected by filtration, washed with water, and recrystallized from MeOH to give the product as a white solid (1.5 g, 80%).

General procedure for obtaining 1,2,4-triazole derivatives **20a**–**20g**

To a suspension of compound **16** (0.25 g, 1.06 mmol) in 1 mL of MeOH was added 0.304 mL of a 3.5 M solution of MeONa in MeOH. The solution obtained was stirred at room temperature for 30 min, and then corresponding bromide was added. The reaction mixture was stirred at 60 °C for 12 h and then the solvent was evaporated under reduced pressure. The product was extracted with Et_2_O. The desired compound was purified using column chromatography using a *n*-hexane to ethyl acetate gradient.

5-(Adamantan-1-yl)-3-((3,7-dimethyloctyl)thio)-1*H*-1,2,4-triazole **20a**

Colorless oil, yield 75%.


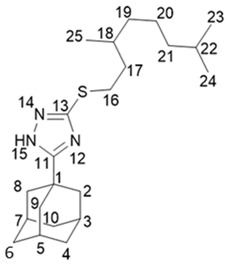


^1^H-NMR (600 MHz, CDCl_3_) (ppm) δ: 0.82 (d, *J*(23, 22) = *J*(24, 22) = 6.7 Hz, 6Н, 3Н-23, 3Н-24), 0.84 (d, *J*(25, 18) = 6.5 Hz, 3Н, Н-25), 1.03–1.12 (m, 3Н, Н-19b, 2Н-21), 1.15–1.29 (m, 3Н, Н-19a, 2Н-20), 1.42–1.55 (m, 3Н, Н-17b, Н-18, Н-22), 1.62–1.69 (m, 1Н, Н-17a), 1.70–1.77 (m, 6Н, 2Н-4, 2Н-6, 2Н-10), 1.96 (d, ^3^*J* = 3.0 Hz, 6Н, 2Н-2, 2Н-8, 2Н-9), 2.02–2.06 (m, 3Н, Н-3, Н-5, Н-7), 3.02 (ddd, *J*(16b, 16a) = 12.5 Hz, *J*(16b, 17b) = 9.5 Hz, *J*(16b, 17a) = 6.3 Hz, 1Н, Н-16b), 3.10 (ddd, *J*(16a, 16b) = 12.5 Hz, *J*(16a, 17a) = 9.8 Hz, *J*(16a, 17b) = 5.4 Hz, 1Н, H-16a). ^13^C-NMR (150 MHz, CDCl_3_) (ppm) δ: 34.12 (s, С-1), 40.68 (t, С-2, С-8,С-9), 27.91 (d, С-3, С-5, С-7), 36.25 (t, С-4, С-6, С-10), 166.49 (s, С-11), 159.05 (s, С-13), 30.24 (t, С-16), 36.58 (t, С-17), 32.10 (d, С-18) 36.76 (t, С-19), 24.49 (t, С-20), 39.08 (t, С-21), 27.81 (d, С-22), 22.45, 22.55 (2q, С-23, С-24), 19.17 (q, С-25). HR MS: 375.2705 (M^+^, C_22_H_37_N_3_S_1_^+^; calc. 375.2703).

5-(Adamantan-1-yl)-3-(((*S*)-3,7-dimethyloct-6-en-1-yl)thio)-1*H*-1,2,4-triazole **20b**

Colorless oil, yield 77%.


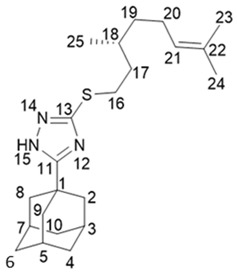


^1^H-NMR (600 MHz, CDCl_3_) (ppm) δ: 0.84 (d, *J*(25, 18) = 6.5 Hz, 3Н, Н-25), 1.07–1.14 (m, 1Н, Н-19b), 1.25–1.32 (m, 1Н, Н-19a), 1.42–1.55 (m, 2Н, Н-17b, Н-18), 1.54 (br.s, 3Н, Н-24), 1.62 (m, 3Н, Н-23), 1.61–1.68 (m, 1Н, Н-17a), 1.68–1.75 (m, 6Н, 2Н-4. 2Н-6, 2Н-10), 1.84–1.98 (m, 2Н, Н-20), 1.94 (d, ^3^*J* = 3.0 Hz, 6Н, 2Н-2, 2Н-8, 2Н-9), 2.00–2.04 (m, 3Н, Н-3, Н-5, Н-7), 3.01 (ddd, *J*(16b, 16a) = 12.4 Hz, *J*(16b, 17) = 9.4 Hz, *J*(16b, 17a) = 6.2 Hz, 1Н, Н-16b), 3.08 (1Н, ddd, *J*(16a, 16b) = 12.4 Hz, *J*(16a, 17a) = 9.8 Hz, *J*(16a, 17b) = 5.4 Hz, H-16a), 5.03 (t.m, *J*(21, 20) = 7.1 Hz, 1Н, Н-21). ^13^C-NMR (150 MHz, CDCl_3_) (ppm) δ: 34.11 (s, С-1), 40.65 (t, С-2, С-8, С-9), 27.90 (d, С-3, С-5, С-7), 36.22 (t, С-4, С-6, С-10), 166.45 (s, С-11), 158.99 (s, С-13), 30.15 (t, С-16), 36.47 (t, С-17), 31.73 (d, С-18), 36.54 (t, С-19), 25.23 (t, С-20), 124.47 (d, С-21), 131.02 (s, С-22), 25.52 (q, С-23), 17.47 (q, С-24), 19.02 (q, С-25). HR MS: 373.2542 (M^+^, C_22_H_35_N_3_S_1_^+^; calc. 373.2546). ${\left[\alpha \right]}_{D}^{24.5}$ = +7 (c 0.48 in МеОН).

5-(Adamantan-1-yl)-3-(((*E*)-3,7-dimethylocta-2,6-dien-1-yl)thio)-1*H*-1,2,4-triazole **20c**

White solid, mp = 106.6–109.8 °С, yield 67%.


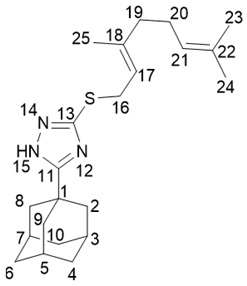


^1^H-NMR (600 MHz, CDCl_3_) (ppm) δ: 1.56 (br.s, 3Н, Н-24), 1.62 (m, 3Н, Н-25), 1.64 (m, 3Н, Н-23), 1.71–1.79 (m, 6Н, 2Н-4, 2Н-6, 2Н-10), 1.93–2.05 (m, 4Н, 2Н-19, 2Н-20), 1.99 (d, ^3^*J* = 3.0 Hz, 6Н, 2Н-2, 2Н-8, 2Н-9), 2.04–2.08 (m, 3Н, Н-3, Н-5, Н-7), 3.72 (d, *J*(16, 17) = 7.8 Hz, 2Н, Н-16), 5.02 (t.m, *J*(20, 21) = 6.9 Hz, 1Н, Н-21), 5.33 (t.m, *J*(17, 16) = 7.8 Hz, 1Н, Н-17). ^13^C-NMR (150 MHz, CDCl_3_) (ppm) δ: 34.18 (s, С-1), 40.78 (t, С-2, С-8, С-9), 27.95 (d, С-3, С-5, С-7), 36.30 (t, С-4, С-6, С-10), 167.05 (s, С-11), 158.32 (s, С-13), 30.69 (t, С-16), 118.91 (d, С-17), 140.41 (s, С-18), 39.43 (t, С-19), 26.27 (t, С-20), 123.74 (d, С-21), 131.64 (s, С-22), 25.55 (q, С-23), 17.59 (q, С-24), 15.98 (q, С-25). HR MS: 371.2389 (M^+^, C_22_H_33_N_3_S_1_^+^; calc. 371.2390).

5-(Adamantan-1-yl)-3-(((*Z*)-3,7-dimethylocta-2,6-dien-1-yl)thio)-1*H*-1,2,4-triazole **20d**

White solid, mp = 110.9–113.5 °С, yield 60%.


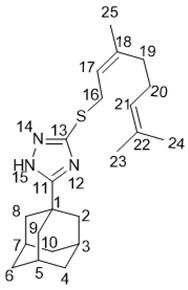


^1^H-NMR (600 MHz, CDCl_3_) (ppm) δ: 1.56 (m, 3Н, Н-24), 1.64 (br.s, 3Н, Н-23), 1.67 (m, 3Н, Н-25), 1.70–1.77 (m, 6Н, 2Н-4, 2Н-6, 2Н-10), 1.96 (d, ^3^*J* = 3.0 Hz, 6Н, 2Н-2, 2Н-8, 2Н-9), 1.98–2.06 (m, 7Н, Н-3, Н-5, Н-7, 2Н-19, 2Н-20), 3.70 (d.m, *J*(16, 17) = 8.0 Hz, 2Н, Н-16), 5.06 (t.m, *J*(21, 20) = 6.7 Hz, 1Н, Н-21), 5.32 (t.m, *J*(17, 16) = 8.0 Hz, 1Н, Н-17). ^13^C-NMR (150 MHz, CDCl_3_) (ppm) δ: 34.14 (s, С-1), 40.71 (t, С-2, С-8, С-9), 27.93 (d, С-3, С-5, С-7), 36.26 (t, С-4, С-6, С-10), 166.70 (s, С-11), 158.63 (s, С-13), 30.40 (t, С-16), 119.60 (d, С-17), 140.32 (s, С-18), 31.71 (t, С-19), 26.48 (t, С-20), 123.72 (d, С-21), 131.79 (s, С-22), 25.53 (q, С-23), 17.54 (q, С-24), 23.27 (q, С-25). HR MS: 371.2393 (M^+^, C_22_H_33_N_3_S_1_^+^; calc. 371.2390).

5-(Adamantan-1-yl)-3-((((*S*)-4-(prop-1-en-2-yl)cyclohex-1-en-1-yl)methyl)thio)-1*H*-1,2,4-triazole **20e**

Colorless oil, yield 65%.


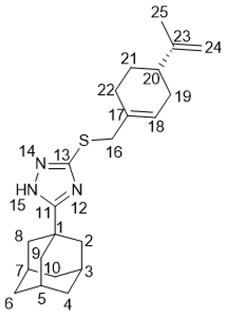


^1^H-NMR (600 MHz, CDCl_3_) (ppm) δ: 1.38–1.47 (m, 1Н, Н-21b), 1.68 (br.s, 3Н, Н-25), 1.71–1.81 (m, 7Н, 2Н-4, 2Н-6, 2Н-10, Н-21a), 1.82–1.90 (m, 1Н, Н-19b), 1.99 (d, ^3^*J* = 3.0 Hz, 6Н, 2Н-2, 2Н-8, 2Н-9), 2.01–2.10 (m, 5Н, Н-3, Н-5, Н-7, Н-19a, Н-20), 2.13–2.18 (m, 2Н, Н-22), 3.65 (br.d, *J*(16b, 16a) = 12.9 Hz, 1Н, Н-16b), 3.69 (br.d, *J*(16a, 16b) = 12.9 Hz, 1Н, Н-16a), 4.64 (m, 1Н, Н-24b), 4.67 (m, 1Н, Н-24a), 5.61–5.65 (m, 1Н, Н-18). ^13^C-NMR (150 MHz, CDCl_3_) (ppm) δ: 34.18 (s, С-1), 40.77 (t, С-2, С-8, С-9), 27.94 (d, С-3, С-5, С-7), 36.28 (t, С-4, С-6, С-10), 166.96 (s, С-11), 158.19 (s, С-13), 39.90 (t, С-16), 133.01 (s, С-17), 125.29 (d, С-18), 30.63 (t, С-19), 40.56 (d, С-20), 27.38, 27.43 (2t, С-21, С-22), 149.50 (s, С-23), 108.56 (t, С-24), 20.64 (q, С-25). HR MS: 369.2235 (M^+^, C_22_H_33_N_3_S_1_^+^; calc. 369.2233). ${\left[\alpha \right]}_{D}^{24}$ = ȡ243 (c 0.24 in МеОН).

5-(Adamantan-1-yl)-3-((((1*R*,5*S*)-6,6-dimethylbicyclo[3.1.1]hept-2-en-2-yl)methyl)thio)-1*H*-1,2,4-triazole **20f**

White solid, mp = 121.1–121.5 °С, yield 70%.


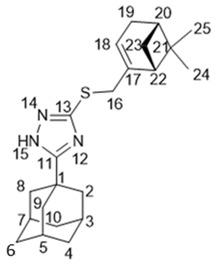


^1^H-NMR (600 MHz, CDCl_3_) (ppm) δ: 0.72 (s, 3Н, Н-25), 1.07 (d, *J*(23b, 23a) = 8.7 Hz, 1Н, Н-23b), 1.23 (s, 3Н, Н-24), 1.70–1.78 (m, 6Н, 2Н-4, 2Н-6, 2Н-10), 1.97 (d, ^3^*J* = 3.0 Hz, 6Н, 2Н-2, 2Н-8, 2Н-9), 2.00–2.09 (m, 4Н, Н-3, Н-5,Н-7, Н-20), 2.14 (d.m, *J*(19b, 19a) = 17.8 Hz, 1Н, Н-19b), 2.17–2.23 (m, 2Н, Н-19a, H-22), 2.34 (1Н, ddd, *J*(23a, 23b) = 8.7 Hz, *J*(23a, 20) = *J*(23a, 22) = 5.6 Hz, Н-23a), 3.66–3.72 (m, 2Н, Н-16), 5.45–5.48 (m, 1Н, Н-18). ^13^C-NMR (150 MHz, CDCl_3_) (ppm) δ: 34.17 (s, С-1), 40.73 (t, С-2, С-8, С-9), 21.93 (d, С-3, С-5, С-7), 36.29 (t, С-4, С-6, С-10), 166.83 (s, С-11), 158.17 (s, С-13), 38.88 (t, С-16), 142.70 (s, С-17), 121.11 (d, С-18), 31.20 (t, С-19), 40.31 (d, С-20), 37.97 (s, С-21), 44.97 (d, С-22), 31.56 (t, С-23), 25.96 (q, С-24), 20.95 (q, С-25). HR MS: 369.2233 (M^+^, C_22_H_31_N_3_S_1_^+^; calc. 369.2234). ${\left[\alpha \right]}_{D}^{24.5}$ = −14 (c 0.53 in МеОН).

5-(Adamantan-1-yl)-3-((2-((1*R*,5*S*)-6,6-dimethylbicyclo[3.1.1]hept-2-en-2-yl)ethyl)thio)-1*H*-1,2,4-triazole **20g**

Colorless oil, yield 85%.


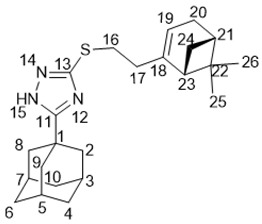


^1^H-NMR (600 MHz, CDCl_3_) (ppm) δ: 0.78 (s, 3Н, Н-26), 1.10 (d, *J*(24b, 24a) = 8.6 Hz, 1Н, Н-24b), 1.22 (s, 3Н, Н-25), 1.69–1.76 (m, 6Н, 2Н-4, 2Н-6, 2Н-10), 1.95 (d, ^3^*J* = 3.0 Hz, 6Н, 2Н-2, 2Н-8, 2Н-9), 1.99–2.05 (m, 5Н, Н-3, Н-5, Н-7, Н-21, Н-23), 2.14 (dm, J(20b, 20a) = 17.6 Hz, 1H, H-20b), 2.20 (dm, *J*(20a, 20b) = 17.6 Hz, 1H, H-20a), 2.25–2.32 (m, 3Н, 2Н-17, Н-24a), 3.02–3.11 (m, 2Н, Н-16), 5.19–5.22 (m, 1Н, Н-19). ^13^C-NMR (150 MHz, CDCl_3_) (ppm) δ: 34.12 (s, С-1), 40.67 (t, С-2, С-8, С-9), 27.90 (d, С-3, С-5, С-7), 36.24 (t, С-4, С-6, С-10), 166.45 (s, С-11), 159.01 (s, С-13), 30.25 (t, С-16), 36.89 (t, С-17), 146.03 (s, С-18), 117.65 (d, С-19), 31.11 (t, С-20), 40.58 (d, С-21), 37.83 (s, С-22), 45.39 (d, С-23), 31.47 (t, С-24), 26.12 (q, С-25), 21.03 (q, С-26). HR MS: 383.2388 (M^+^, C_23_H_33_N_3_S_1_^+^; calc. 383.2390). ${\left[\alpha \right]}_{D}^{24.5}$ = −21 (c 0.51 in МеОН).

General procedure for synthesis of bromides

To a solution of the corresponding monoterpenoid alcohol (6.5 mmol) in 30 mL of Et_2_O cooled to 0 °C was added 0.3 mL (3.1 mmol) of PBr_3_ dropwise. The resulting mixture was stirred at the same temperature for 4 h. After the reaction was completed, the solution was poured onto crushed ice and extracted with Et_2_O. The combined organic layer was washed with saturated aqueous NaHCO_3_ then brine and dried over anhydrous sodium sulfate. The solvent was removed under reduced pressure; the resulting crude was used without further purification. ^1^H NMR spectra were consistent with previously reported data.

(*E*)-1-Bromo-3,7-dimethylocta-2,6-diene 19c, yield 84%. The spectral data were consistent with those in the literature [49].

(*S*)-1-(Bromomethyl)-4-(prop-1-en-2-yl)cyclohex-1-ene **19e**, yield 77%. The spectral data were consistent with those in the literature [50].

2-(Bromomethyl)-6,6-dimethylbicyclo[3.1.1]hept-2-ene **19f**, yield 72%. The spectral data were consistent with those in the literature [51].

A solution of PBr_3_ (0.247 mL, 2.6 mmol) in 4 mL of dry THF was added dropwise to a solution of nerol (1.0 g, 6.5 mmol) in 7 mL of THF under argon at −10 °C. The reaction mixture was stirred for 1.5 h and the solvent was evaporated under reduced pressure. The residue was dissolved in a mixture of *n*-hexane/diethyl ether (1:1). The solution was washed with a saturated aqueous solution of NaHCO_3_, then with water and brine. The organic phase was dried over sodium sulfate, and the solvent was evaporated. The product was used directly without further purification.

(*Z*)-1-Bromo-3,7-dimethylocta-2,6-diene **19d**, yield 68%. The spectral data were consistent with those in the literature [33].

To a solution of PPh_3_ (12.2 g, 46 mmol) in dry DCM (46 mL) was added N-bromosuccinimide (NBS) (8.4 g, 46 mmol) in small portions under an ice-water bath. The mixture was cooled to r.t. and was stirred for 30 min. Then, pyridine (2 mL) was added, followed by the corresponding alcohol (24 mmol). The reaction mixture was stirred overnight at room temperature. Then, the mixture was diluted with *n*-hexane (60 mL) and filtered through a silica gel plug. The reaction flask was stirred three times with EtOAc: *n*-hexane (6:6 mL) for around 1 h and filtered through the silica gel plug. The solution was concentrated in vacuo. The crude residue was purified by flash chromatography (*n*-hexane) to obtain the corresponding bromide as a colorless oil. The ^1^H NMR spectra were consistent with previously reported data.

1-Bromo-3,7-dimethyloctane **19a**, yield 90%. The spectral data were consistent with those in the literature [52].

(*S*)-8-Bromo-2,6-dimethyloct-2-ene **19b**, yield 85%. The spectral data were consistent with those in the literature [53].

2-(2-Bromoethyl)-6,6-dimethylbicyclo[3.1.1]hept-2-ene **19g**, yield 88%. The spectral data were consistent with those in the literature [34].

Synthesis of 5-(adamantan-1-yl)-1,3,4-thiadiazol-2-amine **17**

A solution of 2.2 g (8.7 mmol) of 2-(adamantane-1-carbonyl) hydrazine-1-carbothioamide in 20 mL of concentrated sulfuric acid was kept at room temperature overnight, and then the solution was neutralized with aqueous ammonia solution while cooling with ice until pH 7 was reached. The resulting solid was filtered off, washed with water, dried and recrystallized from EtOH to yield 5-(adamantan-1-yl)-1,3,4-thiadiazol-2-amine as a pale yellow solid (1.46 g, 71%).

Synthesis of 3,7-dimethyloctanoic acid **23a**

A solution of 3,7-dimethyloctan-1-ol (5.0 g, 31.6 mmol) in acetone (17 mL) was added dropwise to a stirred solution of chromium trioxide (7.0 g, 70 mmol) in water (11 mL) and concentrated sulfuric acid (2 mL) cooled to 0 °C for 30 min. The mixture was then diluted with water and extracted with ether. The organic layer was washed with 10% sodium hydroxide solution and the water fraction was separated followed by acidification of the alkaline solution by 10% HCl. The product was extracted with ether to afford 3,7-dimethyloctanoic acid (3.06 g, 55%) as a colorless oil.

Synthesis of (-)-myrtenic acid **23c**

A solution of NaClO_2_ (8.0 g, 70 mmol) in H_2_O (70 mL) was added slowly for 2 h to a stirred mixture of (-)-myrtenal (7.7 g, 51 mmol) in CH_3_CN (50 mL), KH_2_PO_4_ (1.8 g) in water (20 mL), H_2_O_2_ (37%, 5 mL, 52 mmol) and polyethylene glycol (PEG-400, 3.0 g) at 10 °C with ice-water cooling. The reaction was stirred overnight at room temperature. Then, Na_2_SO_3_ (0.5 g) was added. The resulting mixture was acidified with 10% aqueous HCl to pH 3 and extracted several times with diethyl ether. The separated organic phase was washed with saturated sodium bisulfite and water, respectively, and then dried over anhydrous sodium sulfate. Evaporation of the solvent gave (-)-myrtenic acid as a yellow solid (7.67 g, 90%).

Synthesis of (*S*)-4-(prop-1-en-2-yl)cyclohex-1-ene-1-carboxylic acid **23d**

A solution of 1.12 g (10 mmol) of 80% sodium chlorite in 10 mL of distilled water was added dropwise over 30 min at ambient temperature to a stirred mixture of 1.0 g (6.7 mmol) of (*S*)-(-)-perillaldehyde in 7 mL of DMSO and 0.28 g of KH_2_PO_4_ in 3 mL of water. The mixture was stirred at ambient temperature overnight. The reaction mixture was quenched with 30 mL of 10% sodium hydroxide solution followed by extraction of non-acidic compounds with 80 mL of diethyl ether three times. After acidifying the aqueous phase with concentrated HCl, the acid was extracted with 30 mL of diethyl ether three times. The ether layers were combined and dried over sodium sulfate. The evaporation of the solvent under reduced pressure gave 0.5 g (50%) of a slightly yellow solid.

General procedure for acid chloride synthesis

To a solution of carboxylic acid (1.8 mmol) in 10 mL of benzene was added SOCl_2_ (5.4 mmol). The solution was refluxed for 3 h and the solvent was evaporated under reduced pressure. The crude material was used with no further purification.

General procedure for obtaining **25a**–**d** and **26a**

Method A. A solution of corresponding carboxylic acid chloride (1.2 mmol) in dry toluene (1 mL) was added to a suspension of amine **17** (1.06 mmol) and triethylamine (0.177 mL, 1.2 mmol) in 10 mL of dry toluene. The reaction mixture was stirred overnight at room temperature and the solvent was evaporated under reduced pressure. To the residue was added the aqueous solution of KOH, followed by extraction with EtOAc. The product was purified using silica gel column chromatography using an *n*-hexane to ethyl acetate gradient.

Method B. To a mixture of a carboxylic acid (0.9 mmol), amine **17** (1.06 mmol), pyridine (0.264 mL) and EtOAc (0.528 mL) were added T3P (50 wt. % in EtOAc, 2 mmol) while stirring. The reaction mixture was stirred overnight, then water was added. The precipitate formed was washed with water and dried. The product obtained was purified using silica gel column chromatography using an *n*-hexane to ethyl acetate gradient.

*N*-(5-(Adamantan-1-yl)-1,3,4-thiadiazol-2-yl)-3,7-dimethyloctanamide **25a**

White solid, mp = 148.6 °С followed by decomposition, yield 61% (method A), 81% (method B).


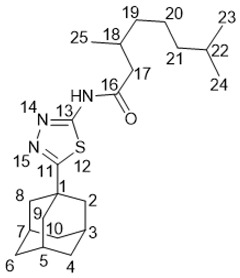


^1^H-NMR (600 MHz, CDCl_3_) (ppm) δ: 0.81 (d, *J*(23, 22) = *J*(24, 22) = 6.7 Hz, 6Н, Н-23, Н-24), 0.97 (d, *J*(25, 18) = 6.7 Hz, 3Н, Н-25), 1.08–1.16 (m, 2Н, Н-21), 1.20–1.43 (m, 4Н, 2Н-19, 2Н-20), 1.43–1.51 (m, 1Н, Н-22), 1.73–1.82 (m, 6Н, 2Н-4, 2Н-6, 2Н-10), 2.06 (d, ^3^*J* = 2.8 Hz, 6Н, 2Н-2, 2Н-8, 2Н-9), 2.07–2.15 (m, 4Н, Н-3, Н-5, Н-7, Н-18), 2.58 (dd, ^2^*J* = 14.1 Hz, *J*(17b, 18) = 8.5 Hz, 1Н, Н-17b), 2.72 (dd, ^2^*J* = 14.1 Hz, *J*(17a, 18) = 6.1 Hz, 1Н, Н-17a). ^13^C-NMR (150 MHz, CDCl_3_) (ppm) δ: 37.71 (s, С-1), 43.07 (t, С-2, С-8, С-9), 28.29 (d, С-3, С-5, С-7), 36.29 (t, С-4, С-6, С-10), 174.22 (s, С-11), 159.87 (s, С-14), 171.69 (s, С-16), 43.72 (t, С-17), 30.95 (d, С-18), 36.88 (t, С-19), 24.37 (t, С-20), 39.04 (t, С-21), 27.76 (d, С-22), 22.41 (q, С-23), 22.56 (q, С-24), 19.28 (q, С-25). HR MS: 389.2495 (M^+^, C_22_H_35_О_1_N_3_S_1_^+^; calc. 389.2496).

*N*-(5-(Adamantan-1-yl)-1,3,4-thiadiazol-2-yl)-3,7-dimethyloct-6-enamide **25b**

White solid, mp = 136.7 °С followed by decomposition, yield 52% (method A), 85% (method B).


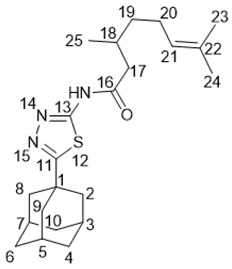


^1^H-NMR (600 MHz, CDCl_3_) (ppm) δ: 0.99 (d, *J*(25, 18) = 6.8 Hz, 3Н, Н-25), 1.26–1.33 (m, 1Н, Н-19b), 1.43–1.50 (m, 1Н, Н-19a), 1.55 (s, 3Н, Н-24), 1.63 (m, 3Н, Н-23), 1.73–1.82 (m, 6Н, 2Н-4, 2Н-6, 2Н-10), 1.92–2.07 (m, 2Н, Н-20), 2.05 (d, ^3^*J* = 2.8 Hz, 6Н, 2Н-2, 2Н-8, 2Н-9), 2.07–2.17 (m, 4Н, Н-3, Н-5, Н-7, Н-18), 2.59 (dd, ^2^*J* = 14.0 Hz, *J*(17b, 18) = 8.5 Hz, 1Н, Н-17b), 2.73 (dd, ^2^*J* = 14.0 Hz, *J*(17a, 18) = 6.1 Hz, 1Н, Н-17a), 5.06 (t.m, *J*(21, 20) = 7.0 Hz, 1Н, Н-21). ^13^C-NMR (150 MHz, CDCl_3_) (ppm) δ: 37.74 (s, С-1), 43.04 (t, С-2, С-8, С-9), 28.29 (d, С-3, С-5, С-7), 36.26 (t, С-4, С-6, С-10), 174.27 (s, С-11), 159.90 (s, С-14), 171.59 (s, С-16), 43.58 (t, С-17), 30.66 (d, С-18), 36.68 (t, С-19), 25.20 (t, С-20), 124.27 (d, С-21), 131.25 (s, С-22), 25.56 (q, С-23), 17.50 (q, С-24), 19.21 (q, С-25). HR MS: 387.2339 (M^+^, C_22_H_33_О_1_N_3_S_1_^+^; calc. 387.2337).

(1*R*,5*S*)-*N*-(5-(Adamantan-1-yl)-1,3,4-thiadiazol-2-yl)-6,6-dimethylbicyclo[3.1.1]hept-2-ene-2-carboxamide **25c**

White solid, mp = 240.2 °С followed by decomposition, yield 50% (method A), 70% (method B).


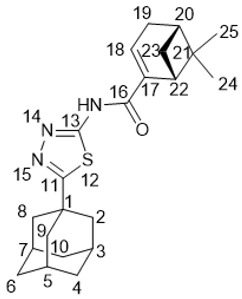


^1^H-NMR (600 MHz, CDCl_3_) (ppm) δ: 0.84 (s, 3Н, Н-25), 1.21 (d, *J*(23b, 23a) = 9.2 Hz, 1Н, Н-23b), 1.34 (s, 3Н, Н-24), 1.72–1.81 (m, 6Н, 2Н-4, 2Н-6, 2Н-10), 2.05 (d, ^3^*J* = 2.7 Hz, 6Н, 2Н-2, 2Н-8, 2Н-9), 2.07–2.11 (m, 3Н, Н-3, Н-5, Н-7), 2.14–2.19 (m, 1Н, Н-20), 2.47–2.53 (2Н, m, Н-19b, Н-23a), 2.56 (ddd, ^2^*J* = 19.7 Hz, *J*(19a, 18) = *J*(19a, 20) = 3.0 Hz, 1Н, Н-19a), 2.91 (ddd, J(22, 20) = *J*(22, 23a) = 5.6 Hz, *J*(22, 18) = 1.5 Hz, 1Н, Н-22), 7.13–7.16 (m, 1Н, Н-18). ^13^C-NMR (150 MHz, CDCl_3_) (ppm) δ: 37.68 (s, С-1), 43.07 (t, С-2, С-8, С-9), 28.28 (d, С-3, С-5, С-7), 36.29 (t, С-4, С-6, С-10), 173.99 (s, С-11), 160.51 (s, С-14), 164.92 (s, С-16), 140.86 (s, С-17), 136.01 (d, С-18), 32.33 (t, С-19), 40.16 (d, С-20), 37.83 (s, С-21), 41.17 (d, С-22), 31.23 (t, С-23), 25.76 (q, С-24), 20.93 (q, С-25). HR MS: 383.2026 (M^+^, C_22_H_29_О_1_N_3_S_1_^+^; calc. 383.2024). ${\left[\alpha \right]}_{D}^{26}$ = −11 (c 0.84 in CHCl_3_).

(*S*)-*N*-(5-(adamantan-1-yl)-1,3,4-thiadiazol-2-yl)-4-(prop-1-en-2-yl)cyclohex-1-ene-1-carboxamide **25d**

White solid, mp = 265.6 °С followed by decomposition, yield 55% (method A), 75% (method B).


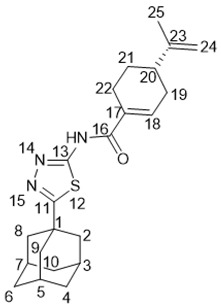


^1^H-NMR (600 MHz, CDCl_3_) (ppm) δ: 1.49–1.57 (m, 1Н, Н-21b), 1.72–1.82 (m, 6Н, 2Н-4, 2Н-6, 2Н-10) 1.75 (s, 3Н, Н-25), 1.92–1.98 (m, 1Н, Н-21a), 2.06 (d, ^3^*J* = 3.0 Hz, 6Н, 2Н-2, 2Н-8, 2Н-9), 2.07–2.12 (m, 3Н, Н-3, Н-5, Н-7), 2.18–2.27 (m, 2Н, Н-19b, H-20), 2.35–2.44 (m, 1H, H-22b), 2.45–2.52 (m, 1H, H-19a), 2.58–2.65 (m, 1H, H-22a), 4.72 (br.s, 1Н, Н-24b), 4.75–4.77 (m, 1Н, Н-24a), 7.23–7.26 (m, 1Н, Н-18). ^13^C-NMR (150 MHz, CDCl_3_) (ppm) δ: 37.81 (s, С-1), 42.99 (t, С-2, С-8, С-9), 28.23 (d, С-3, С-5, С-7), 36.21 (t, С-4, С-6, С-10), 174.17 (s, С-11), 160.77 (s, С-14), 166.07 (s, С-16), 130.80 (s, С-17), 139.50 (d, С-18), 31.21 (t, С-19), 39.83 (d, С-20), 26.80 (t, С-21), 24.30 (t, С-22), 148.45 (s, С-23), 109.25 (t, С-24), 20.60 (q, С-25). HR MS: 383.2026 (M^+^, C_22_H_29_О_1_N_3_S_1_^+^; calc. 383.2022) ${\left[\alpha \right]}_{D}^{27}$ = −32 (c 0.74 in CHCl_3_).

N-(Adamantan-1-yl)-3,7-dimethyloctanamide **26a**

White solid, mp = 97.9 °C followed by decomposition, yield 52% (method B).


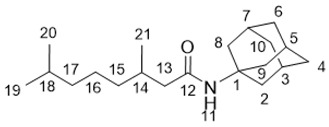


^1^H-NMR (600 MHz, CDCl_3_) (ppm) δ: 0.827 and 0.829 (2d, *J*(19, 18) = *J*(20, 18) = 6.6 Hz, 3H each, H-19, H-20), 0.88 (d, *J*(21, 14) = 6.6 Hz, 3H, H-21), 1.05–1.16 (m, 3H, H-15b, 2H-17), 1.17–1.33 (m, 3H, H-15a, 2H-16), 1.43–1.53 (m, 1H, H-18), 1.60–1.68 (m, 6H, 2H-4, 2H-6, 2H-10), 1.78 (dd, ^2^*J* = 13.6 Hz, *J*(13, 14) = 8.3 Hz, 1H, H-13b), 1.84–1.93 (m, 1H, H-14), 1.94–1.99 (m, 6H, 2H-2, 2H-8, 2H-9), 2.01–2.06 (m, 3H, H-3, H-5, H-7), 2.05 (dd, ^2^*J* = 13.6 Hz, *J*(13a, 14) = 6.0 Hz, 1H, H-13a), 5.08 (br. s, 1H, H-11). ^13^C-NMR (150 MHz, CDCl_3_) (ppm) δ: 51.65 (s, C-1), 41.60 (t, C-2, C-8, C-9), 29.33 (d, C-3, C-5, C-7), 36.27 (t, C-4, C-6, C-10), 171.69 (s, C-12), 45.56 (t, С-13), 30.72 (d, С-14), 36.90 (t, С-15), 24.57 (t, С-16), 38.99 (t, С-17), 27.81 (d, С-18), 22.45, 22.54 (2q, С-19, С-20), 19.46 (q, С-21). HR MS: 305.2715 (M^+^, C_20_H_35_O_1_N_1_^+^; calc. 305.2713).

### 3.2. Biology

#### 3.2.1. Detection of Tdp1 Activity

The recombinant Tdp1 was purified to homogeneity by chromatography on Ni-chelating resin and phosphocellulose P11 as described [54], using plasmid pET 16B-Tdp1, kindly provided by Dr. K.W. Caldecott (University of Sussex, United Kingdom). Tdp1 activity was detected as described in the work [41]. Briefly, we measured the fluorescence intensity after quencher removal from a fluorophore quencher–coupled DNA oligonucleotide catalyzed by Tdp1. The reaction was carried out at different concentrations of inhibitors. The control samples contained 1% DMSO. The reaction mixtures contained Tdp1 buffer (50 mM Tris-HCl pH 8.0, 50 mM NaCl, and 7 mM β-mercaptoethanol), 50 nM biosensor, and an inhibitor being tested. The reaction was started by the addition of purified Tdp1 (1.5 nM). The biosensor (5′-[FAM] AAC GTC AGGGTC TTC C [BHQ]-3′) was synthesized in the Laboratory of Biomedical Chemistry at the Institute of Chemical Biology and Fundamental Medicine (Novosibirsk, Russia).

The reactions were incubated on a POLARstar OPTIMA fluorimeter (BMG LAB-TECH, GmbH) to measure fluorescence every 55 s (ex. 485 nm/em. 520 nm) during the linear phase (here, data are from minute 0 to minute 8). The values of IC_50_ were determined using a six-point concentration response curve in a minimum of three independent experiments, and were calculated using MARS Data Analysis 2.0 (BMG LABTECH).

#### 3.2.2. Cytotoxicity Assays

The human cervical cancer HeLa cells were grown in Iscove’s modified Dulbecco’s medium (IMDM) with 40 μg/mL gentamicin, 50 IU/mL penicillin, 50 μg/mL streptomycin (MP Biomedicals), and 10% of fetal bovine serum (Biolot) in a 5% CO_2_ atmosphere. Human colon adenocarcinoma HCT-116, rectum adenocarcinoma SW837, and human embryo kidney HEK293A cell lines were cultured in DMEM medium (Invitrogen) supplemented with 10% fetal bovine serum (FBS) (Invitrogen), penicillin (100 units/mL), and streptomycin (100 μg/mL), at 37 °C, 5% CO_2_ in a humid atmosphere. After the formation of a 30–50% monolayer, the tested compounds were added to the medium. The volume of the added reagents was 1/100 of the total volume of the culture medium, and the amount of DMSO was 1% of the final volume. Control cells were grown in the presence of 1% DMSO. The cell culture was monitored for 3 days.

The cytotoxicity of the compounds to HeLa and HEK293A cell lines was examined using the EZ4U Cell Proliferation and Cytotoxicity Assay (Biomedica, Austria), according to the manufacturer’s protocols.

The inhibition of HCT-116 and SW837 cell growth was assessed by the MTT test, based on the reduction of MTT (3-(4,5-dimethylthiazol-2-yl)-2,5-diphenyltetrazolium bromide into formazan by mitochondrial NAD(P)H-dependent oxidoreductase enzymes [55]. Cells in the exponential growth phase were seeded in 96-well plates (2000 cells per well). The cells were allowed to attach for 24 h and were treated with compounds with concentrations ranging from 1 to 100 μM for 72 h at 37 °C. MTT solution was added to each well at a final concentration of 0.5 mg/mL, and the plates were incubated at 37 °C for 2 h. The medium was removed, and the dark blue crystals of formazan were dissolved in 100 μL of isopropanol. The absorbance at 570 nm (peak) and 620 nm (baseline) was determined using a microplate reader Multiscan FC (Thermo Fisher Scientific Corporation). All concentrations were performed in triplicate. The values are given as mean ± standard deviation (S.D.) values, and all measurements were repeated three times.

### 3.3. Molecular Modeling and Screening

The compounds were docked against the crystal structure of Tdp1 (PDB ID: 6DIE, resolution 1.78 Å) [43], which was obtained from the Protein Data Bank (PDB) [56,57]. The Scigress version FJ 2.6 program [58] was used to prepare the crystal structure for docking, i.e., the hydrogen atoms were added, and the co-crystallized ligand benzene-1,2,4-tricarboxylic acid was removed, as well were the crystallographic water molecules except HOH 814, 821 and 1078. The waters were toggle-bound or were displaced by the ligand during docking, and spin–automatic optimization of the orientation of the hydrogen atoms was performed. The Scigress software suite was also used to build the inhibitors and the MM2 [59] force field was applied to identify the global minimum using the CONFLEX method [60] followed by structural optimization. The docking center was defined as the position of a carbon on the ring of the co-crystallized benzene-1, 2, 4-tricarboxylic acid (*x* = −6.052, *y* = −14.428, *z* = 33.998) with 10 Å radius. Fifty docking runs were allowed for each ligand with default search efficiency (100%). The basic amino acids lysine and arginine were defined as protonated. Furthermore, aspartic and glutamic acids were assumed to be deprotonated. The GoldScore(GS) [61] and ChemScore (CS) [62,63] ChemPLP (piecewise linear potential) [64] and ASP (Astex statistical potential) [65] scoring functions were implemented to predict the binding modes and relative energies of the ligands using the GOLD v5.4.1 software suite.

The QikProp 6.2 [66] software package was used to calculate the molecular descriptors of the molecules. The reliability was established by QikProp for the calculated descriptors [67]. The known drug indexes (KDI) were calculated from the molecular descriptors as described by Eurtivong and Reynisson [48]. For application in Excel, columns for each property were created and the following equations used to derive the KDI numbers for each descriptor: KDI MW: = EXP(−((MW − 371.76)^2)/((2 × 112.76)^2)); KDI Log P: = EXP(−((LogP − 2.82)^2)/((2 × 2.21)^2)); KDI HD: = EXP(−((HD − 1.88)^2)/((2 × 1.7)^2)); KDI HA: = EXP(−((HA − 5.72)^2)/((2 × 2.86)^2)); KDI RB = EXP(−((RB − 4.44)^2)/((2 × 3.55)^2)), and KDI PSA: = EXP(−((PSA − 79.4)^2)/((2 × 54.16)^2)). These equations could simply be copied into Excel and the descriptor name (e.g., MW) substituted with the value in the relevant column. In order to derive KDI_2A_, this equation was used: = (KDI MW + KDI LogP + KDI HD + KDI HA + KDI RB + KDI PSA) and for KDI_2B_ we used = (KDI MW × KDI LogP × KDI HD × KDI HA × KDI RB × KDI PSA).

## 4. Conclusions

New hybrid molecules consisting of adamantane, monoterpene and heterocyclic fragments were synthesized and tested for their Tdp1-inhibitory properties. All the compounds were found to exhibit an inhibitory activity, and some had submicromolar potency, which was supported by the plausible binding modes in the catalytic pocket predicted by molecular modeling. The triazole and thiadiazole linkers showed marked improvement in potency as compared to the amides and thioamides previously reported [25,40]. The triazole and thiadiazole derivatives showed comparable IC_50_ values and the saturated aliphatic chain, 3,7-dimethyloctane, was the best substituent for both series (**20a**, **25a**). It was found that some of the new ligands potentiated the cytotoxic potential of topotecan in vitro, **20c** being the most effective sensitizer, tripling (3×) the potency. The comparison of substances synthesized with structurally similar compounds **26a**–**c**, **28a**–**g** and **29a** demonstrated that the incorporation of a 1,3,4-thiadiazole core into amides **26a**–**c** increased Tdp1 inhibitory properties. The replacement of the amide bond in **28a**–**c** or the thioamide bond in **29a** with a 1,2,4-triazole linker also led to enhanced activity against Tdp1.

## Figures and Tables

**Table 1:** The results for the Tdp1 activity inhibition at 50% (IC_50_) values of the derivatives. Additionally, the CC_50_ (50% cytotoxic concentration) values are given for cervical cancer (HeLa) and colorectal (HCT-116, SW837) human cancer cell lines.

Structure		IC_50_, μM	CC_50_, μM HeLa	CC_50_, μM HCT-116	CC_50_, μM SW837
**1,2,4-Triazole derivatives** 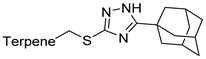					
**20a**	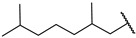	0.54 ± 0.09	85 ± 23	27 ± 6	52 ± 6
**20b**	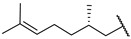	1.5 ± 0.3	>100	>100	41 ± 4
**20c**	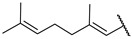	5.3 ± 1.7	64 ± 6	16 ± 7	41 ± 6
**20d**	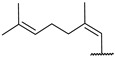	5.6 ± 0.6	31± 11	20 ± 9	17 ± 5
**20e**	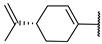	6.2 ± 2.2	24 ± 16	15 ± 5	40 ± 4
**20f**	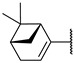	7.5 ± 1.8	15 ± 2	54 ± 5	46 ± 6
**20g**	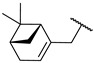	0.57 ± 0.14	74 ± 17	34 ± 6	40 ± 4
**1,3,4-Thiadiazole derivatives** 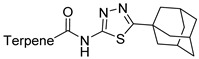					
**25a**	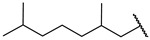	0.35 ± 0.05	>100	>100	>100
**25b**	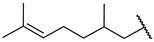	2.59 ± 0.48	>100	66 ± 6	83 ± 8
**25c**	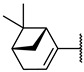	0.45 ± 0.09	>100	>100	>100

**Table 2:** The effect of the Tdp1 inhibitors at 5 µM on the 50% cytotoxic concentration (CC_50_) value of topotecan for HeLa cancer cells. The concentrations of the Tdp1 inhibitors are 5 µM for HeLa cells and ranged from 15 to 45 µM (concentration is given in parenthesis in µM) for the HCT-116 cancer cell line.

Compound	HeLa—CC_50_, μM	HCT116—CC_50_, nM
**Topotecan**	6.0	715.0
**20a**	4.5	650.0 (20)
**20b**	ND *	575.3 (20)
**20c**	1.9	460.0 (15)
**20d**	5.4	689.0 (15)
**20e**	ND	392.0 (15)
**20f**	2.8	715.0 (25)
**20g**	2.6	235.8 (20)
**25a**	4.9	455.7 (45)
**25b**	2.9	611.9 (20)
**25c**	3.6	486.0 (25)

* Not determined due to poor solubility.

## Data Availability

The data presented in this study are available on request from the corresponding author.

## Supplementary Materials

The following are available online. Figure S1: The molecular structures of pharmaceuticals containing 1,2,4-triazole or 1,3,4-thiadiazole moieties, Figure S2: Dose-dependent impact of the Tdp1 inhibitors on the viability of HeLa (**a**), HCT-116 (**b**), and SW837 (**c**) cells, Figure S3: Combination index plot determined for combined action of **20g** and topotecan on HCT-116 cells, Figure S4a: The correlation of the IC50 values of the ligands **20a**, **20b** and **20c** with log P, Figure S4b: The correlation of the IC50 values of the ligands with MW, Figures S5–S40: NMR and mass spectra of the ligands, Table S1: The binding affinities as predicted by the scoring functions used, Table S2: The molecular descriptors and their corresponding Known Drug Indexes 2a and 2b (KDI2a/2b). Table S3: Definition of lead-like, drug-like and Known Drug Space (KDS) in terms of molecular descriptors.

## Sample Availability

Samples of the compounds are available from the authors.

## Abbreviations

Tdp1	Tyrosyl-DNA phosphodiesterase 1
DMSO	Dimethyl sulfoxide
