# Supplementary material for: Novel Tdp1 Inhibitors Based on Adamantane Connected with Monoterpene Moieties via Heterocyclic Fragments
Source: Molecules. 2021 May 24;26(11):3128. doi: 10.3390/molecules26113128 (PMC8197275; doi:10.3390/molecules26113128)
Supplement: Supplementary file 1 [file molecules-26-03128-s001.zip › molecules-1211471-supplementary.pdf]

Novel Tdp1 inhibitors based on adamantane connected with monoterpene moieties via heterocyclic fragments

Aldar A. Munkuev [1], Evgenii S. Mozhaitsev [1], Arina A. Chepanova[2], Evgeniy V. Suslov [1], Dina V. Korchagina [1], Olga D. Zakharova[2], Ekaterina S. Ilina[2], Nadezhda S. Dyrkheeva[2], Alexandra L. Zakharenko [2], Jóhannes Reynisson [3*], Konstantin P. Volcho [1], Nariman F. Salakhutdinov [1] and Olga I. Lavrik [2]

[1] N.N. Vorozhtsov Novosibirsk Institute of Organic Chemistry, Siberian Branch of Russian Academy of Sciences, 9, Lavrentiev Ave., Novosibirsk 630090, Russia
[2] Institute of Chemical Biology and Fundamental Medicine, Siberian Branch of Russian Academy of Sciences, 8, Lavrentiev Ave., Novosibirsk 630090, Russia
[3] School of Pharmacy and Bioengineering, Keele University, Hornbeam Building, Staffordshire ST5 5BG, UK
*Correspondence: j.reynisson@keele.ac.uk; Tel.: +44-(0)-178-2733-985

Supporting Information

Table of Contents

**Figure S1**	The molecular structures of pharmaceuticals containing 1,2,4-triazole or 1,3,4-thiadiazole moieties.	Page 2
**Figure S2**	Dose-dependent impact of the Tdp1 inhibitors on the viability of HeLa (a), HCT-116 (b), and SW837 (c) cells.	Pages 2-3
**Figure S3**	Combination index plot determined for combined action of 20g and topotecan on HCT-116 cells.	Page 3
**Figure S4a**	The correlation of the IC_{50} values of the ligands **20a**, **20b** and **20c** with log P.	Page4
**Figure S4b**	The correlation of the IC_{50} values of the ligands with MW.	Page 4
**Figures S5 - S40**	NMR and mass spectra of the ligands	Pages 5 23
**Table S1**	The binding affinities as predicted by the scoring functions used.	Page 24
**Table S2**	The molecular descriptors and their corresponding Known Drug Indexes 2a and 2b ($KDI_{2a/2b}$). The R^2 numbers derived do not contain the IC_{50} > 15 μM values.	Page 25
**Table S3**	Definition of lead-like, drug-like and Known Drug Space (KDS) in terms of molecular descriptors. The values given are the maxima for each descriptor for the volumes of chemical space used.	Page 25

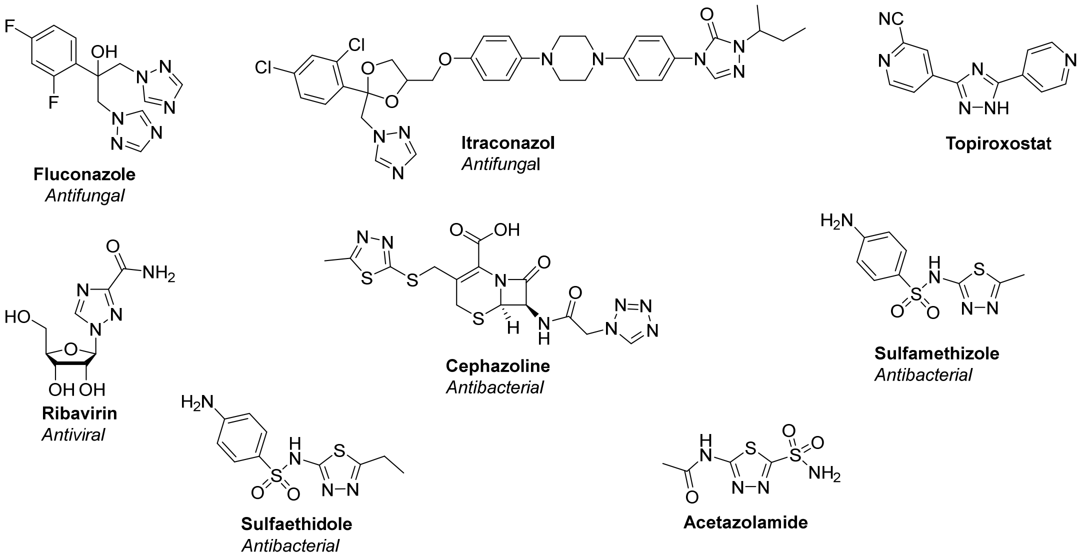

Figure S1. The molecular structures of pharmaceuticals containing 1,2,4-triazole or 1,3,4-thiadiazole moieties.

a)

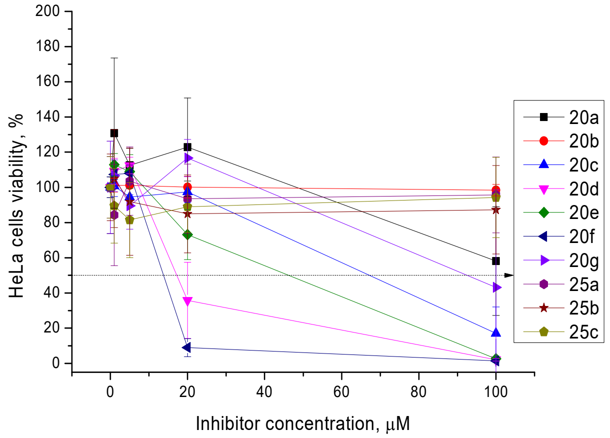

b)

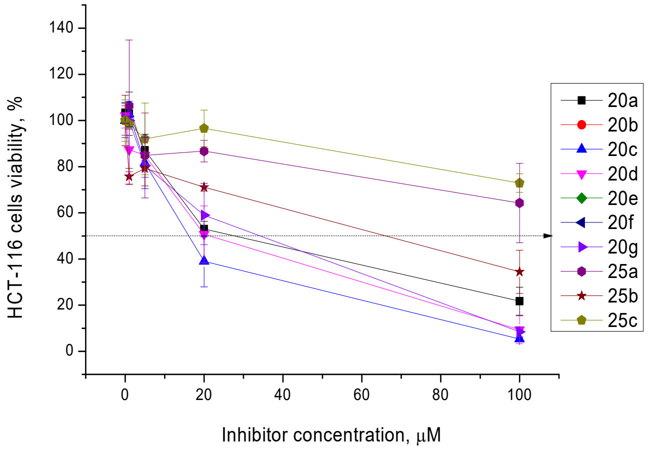

c)

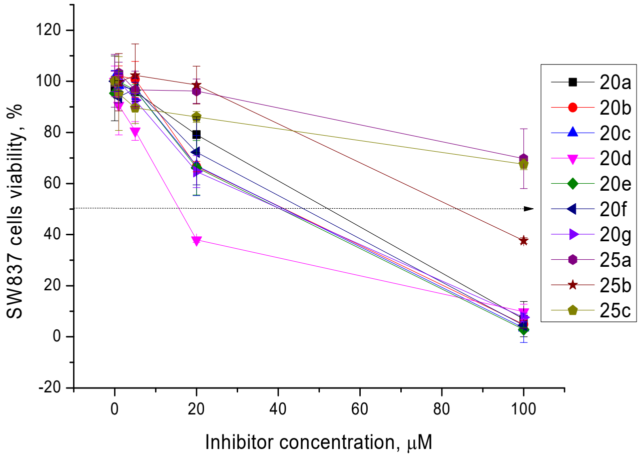

Figure S2. Dose-dependent impact of the Tdp1 inhibitors on the viability of HeLa (a), HCT-116 (b), and SW837 (c) cells.

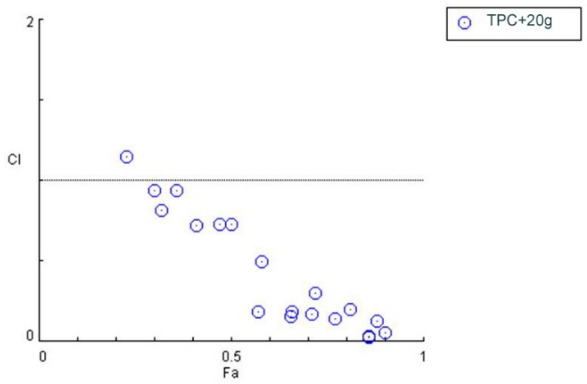

Figure S3. Combination index plot determined for combined action of 20g and topotecan on HCT-116 cells.

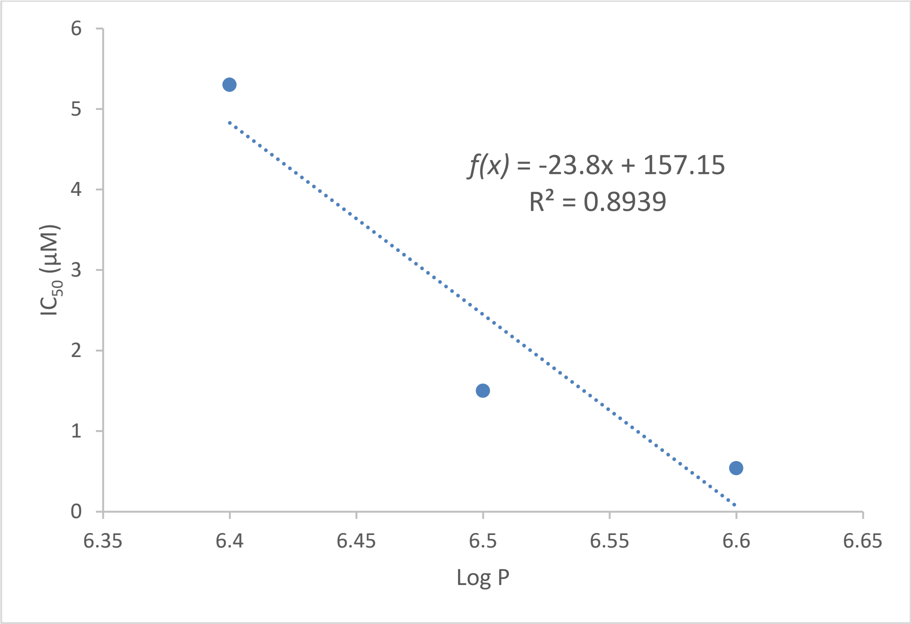

A

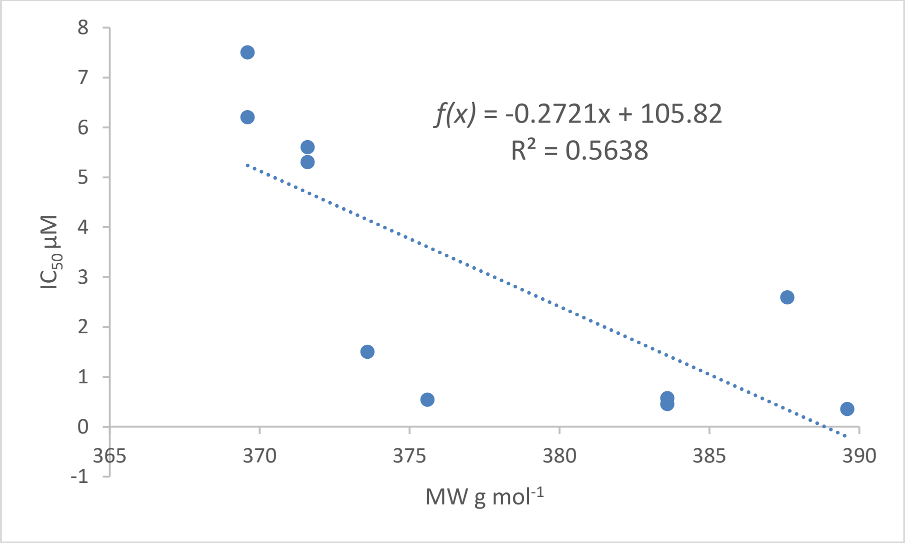

B

Figure S4. A) The correlation of the IC_{50} values of the ligands **20a**, **20b** and **20c** with log P. B) The correlation of the IC_{50} values of the ligands with MW.

Figure S5:^{1}H NMR of compound **20a**

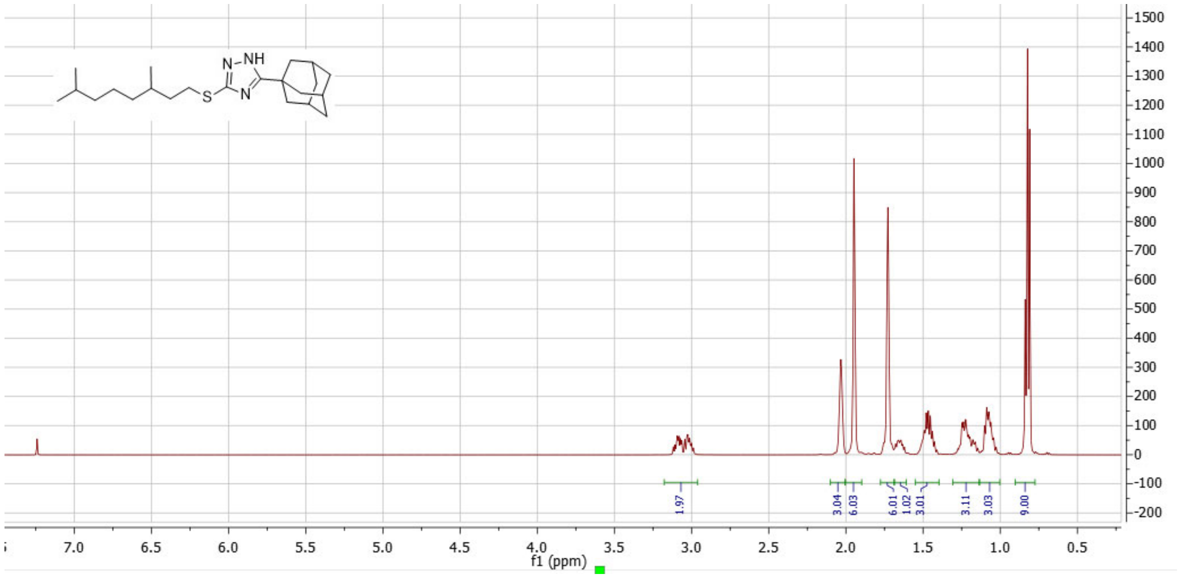

Figure S6:^{13}C NMR of compound **20a**

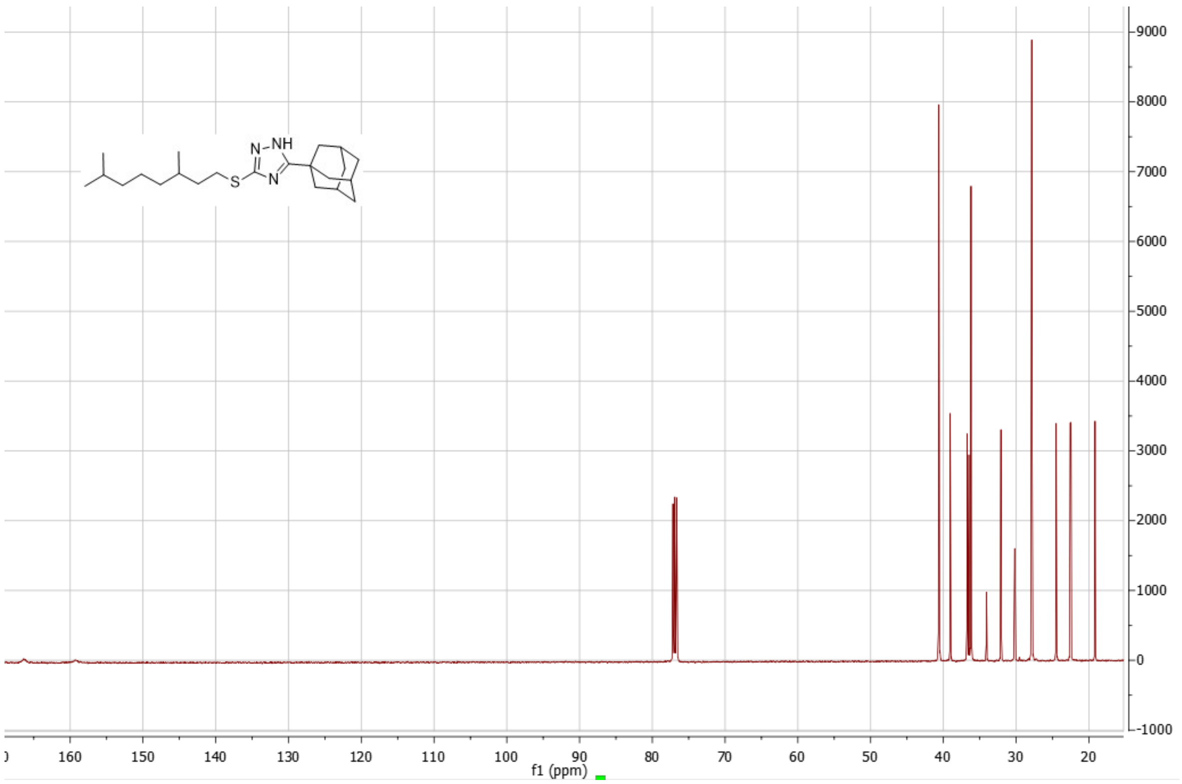

Figure S7: DFS spectrum of compound **20a**

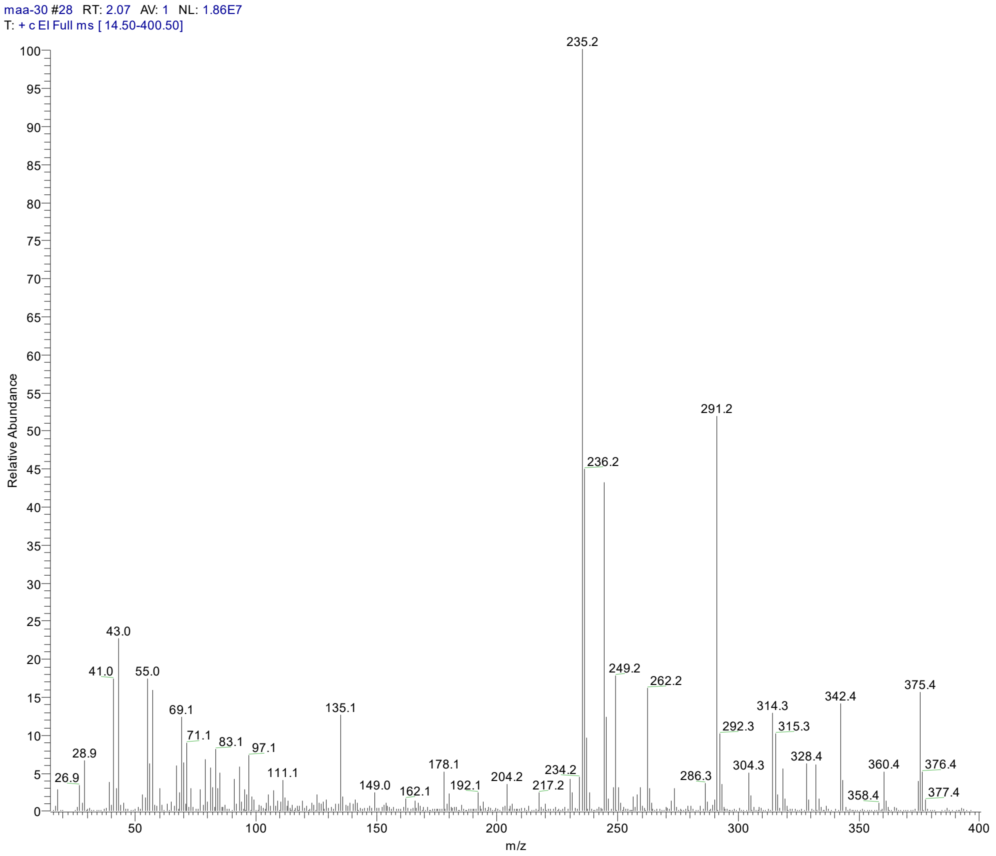

Figure S8: ^{1}H NMR of compound **20b**

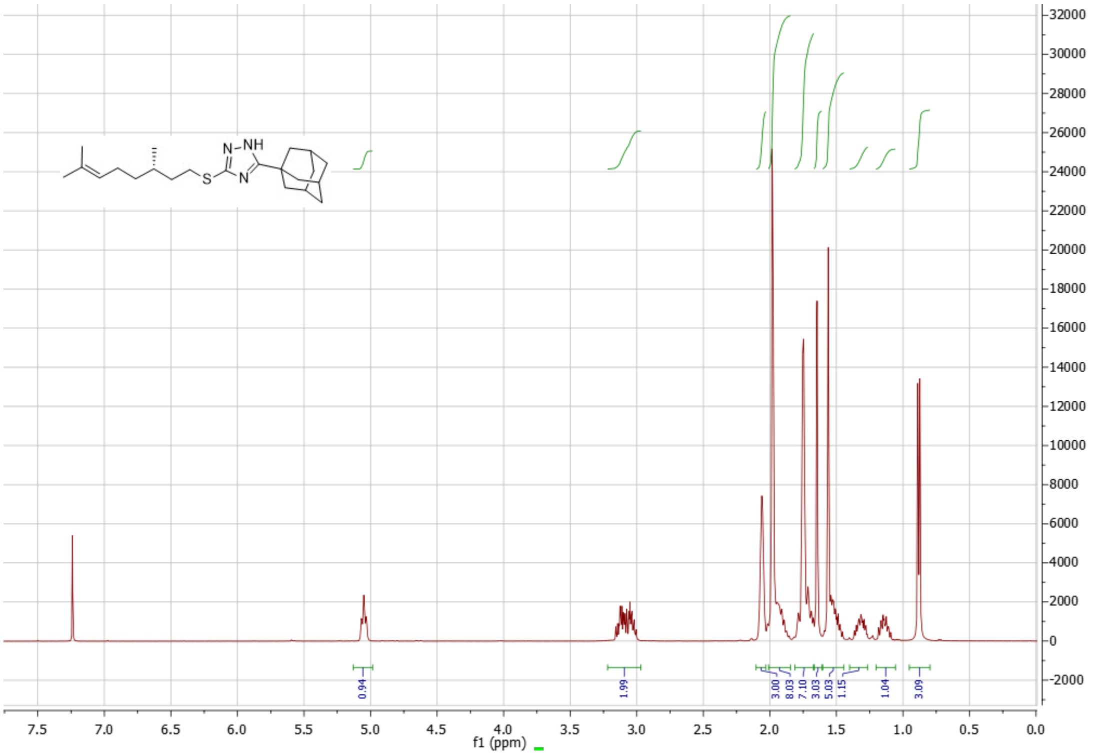

Figure S9: ^{13}C NMR of compound **20b**

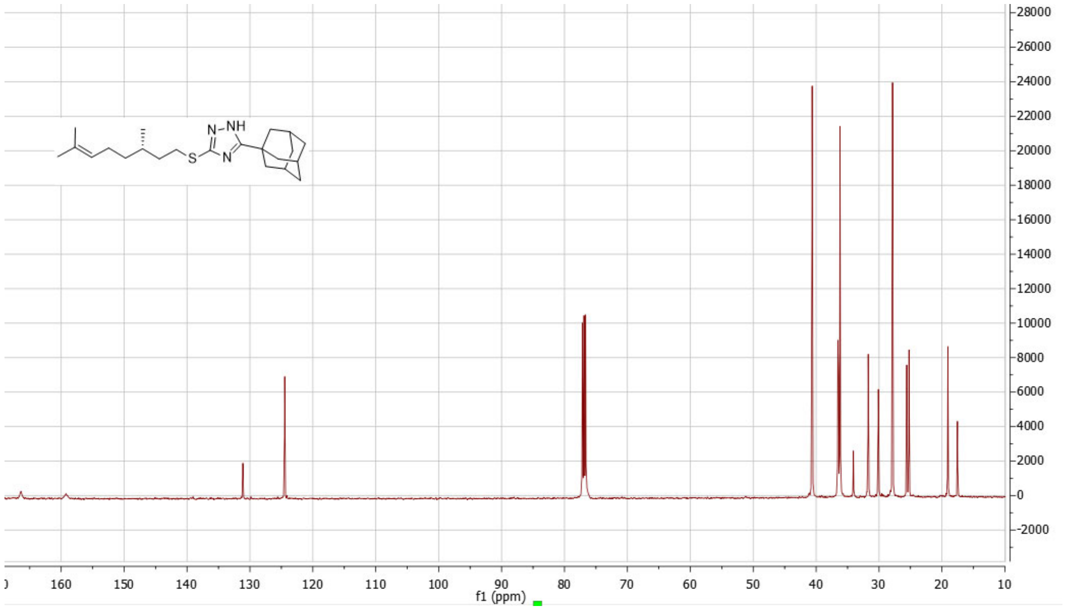

Figure S10: DFS spectrum of compound **20b**

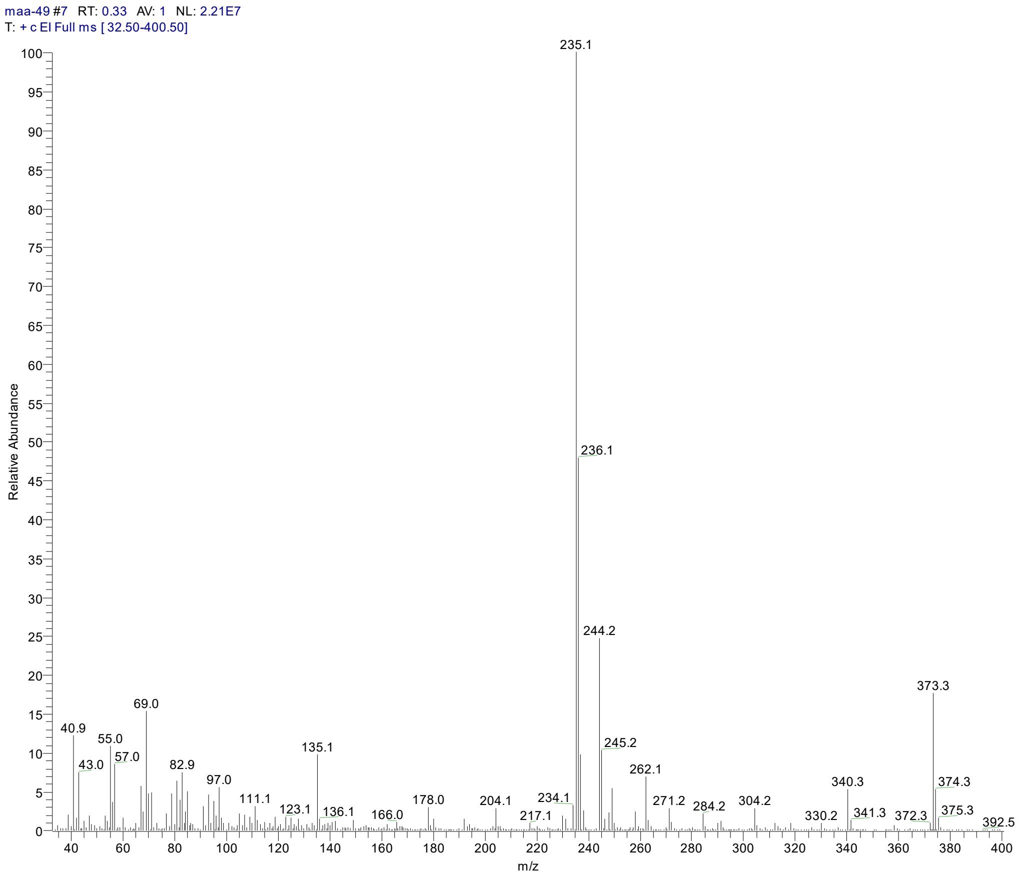

Figure S11: ^{1}H NMR of compound **20c**

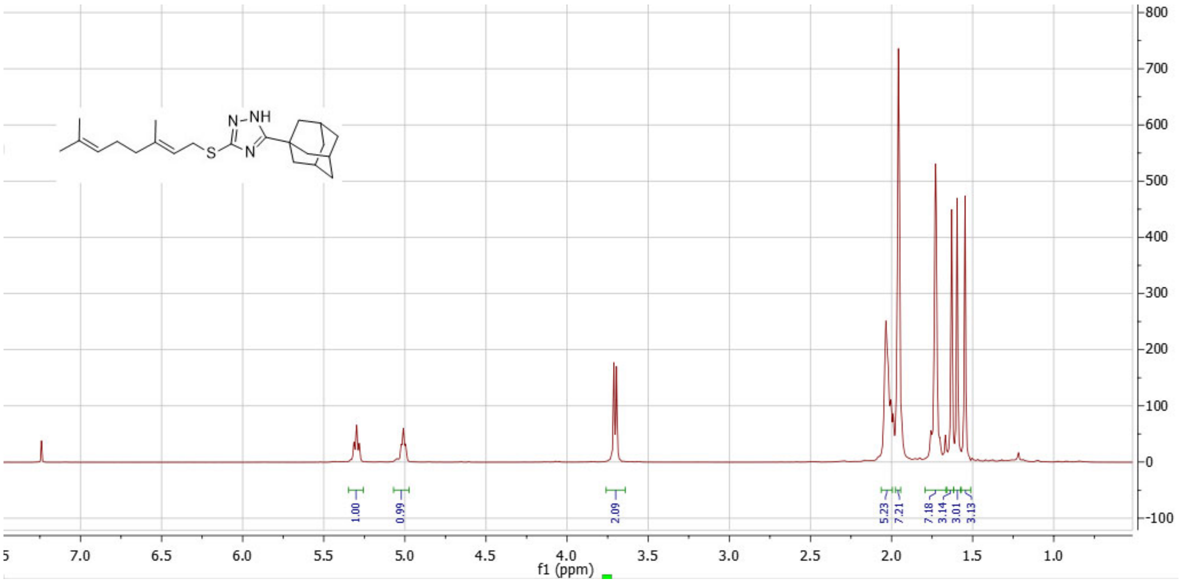

Figure S12: ^{13}C NMR of compound **20c**

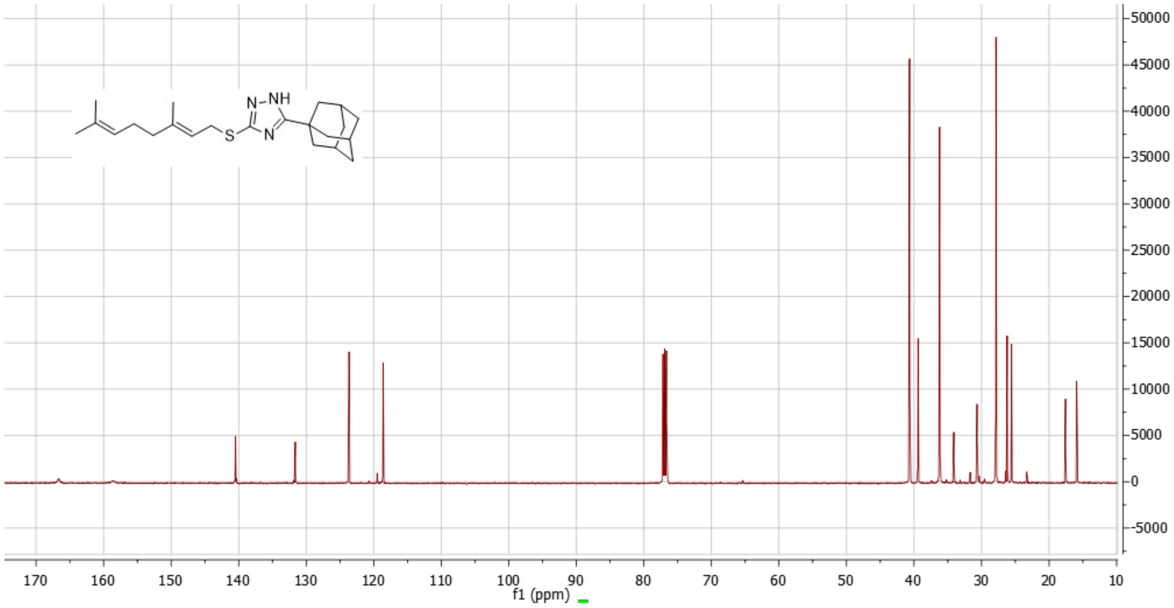

Figure S13: DFS spectrum of compound **20c**

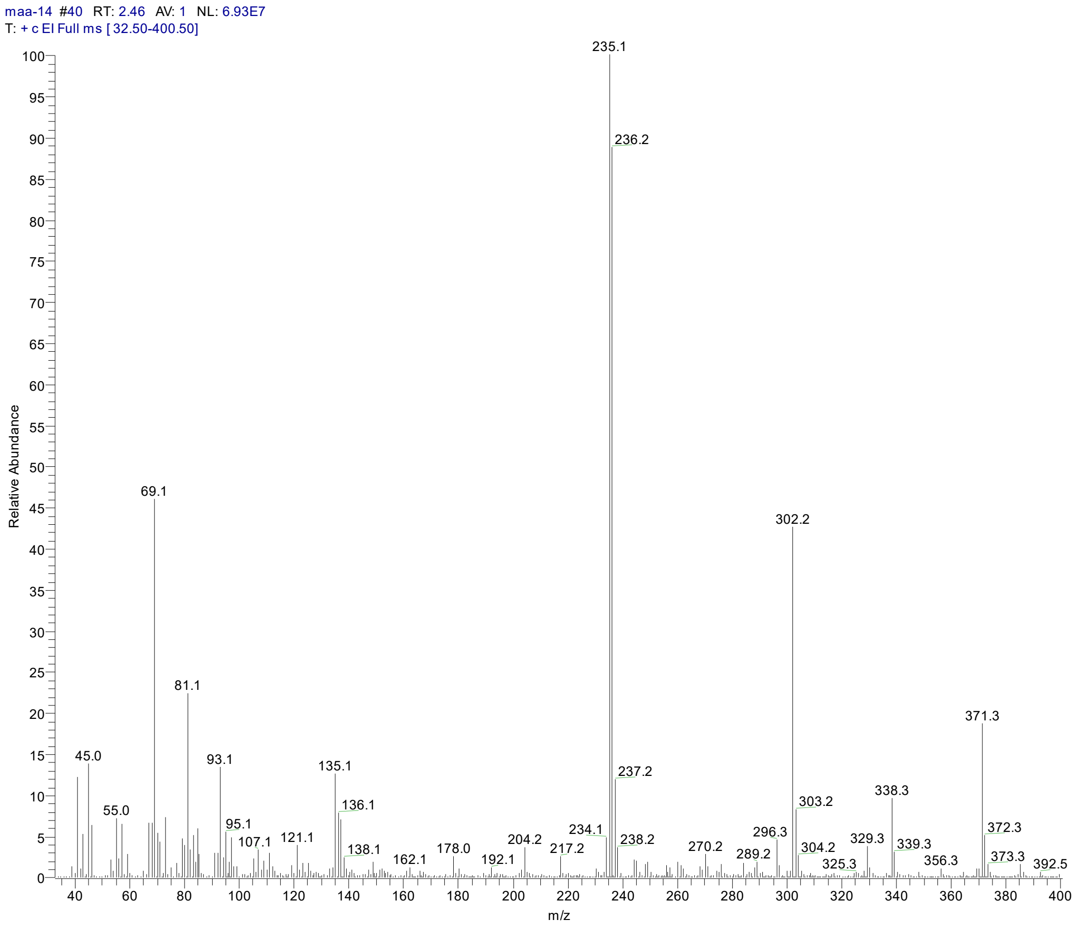

Figure S14: ^{1}H NMR of compound **20d**

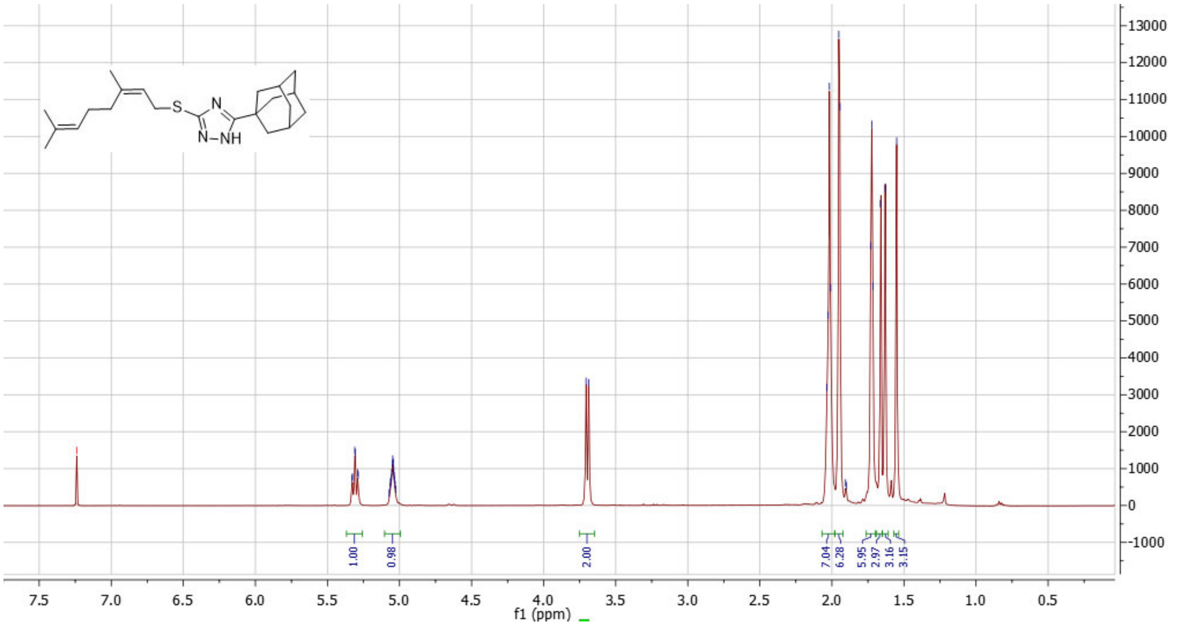

Figure S15: ^{13}C NMR of compound **20d**

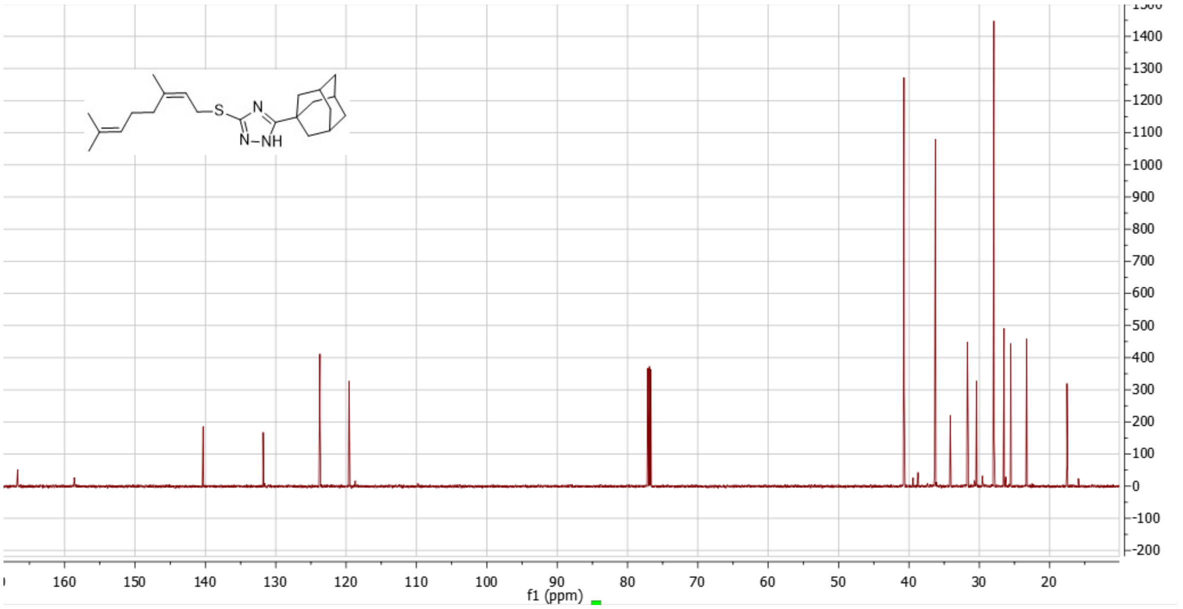

Figure S16: DFS spectrum of compound **20d**

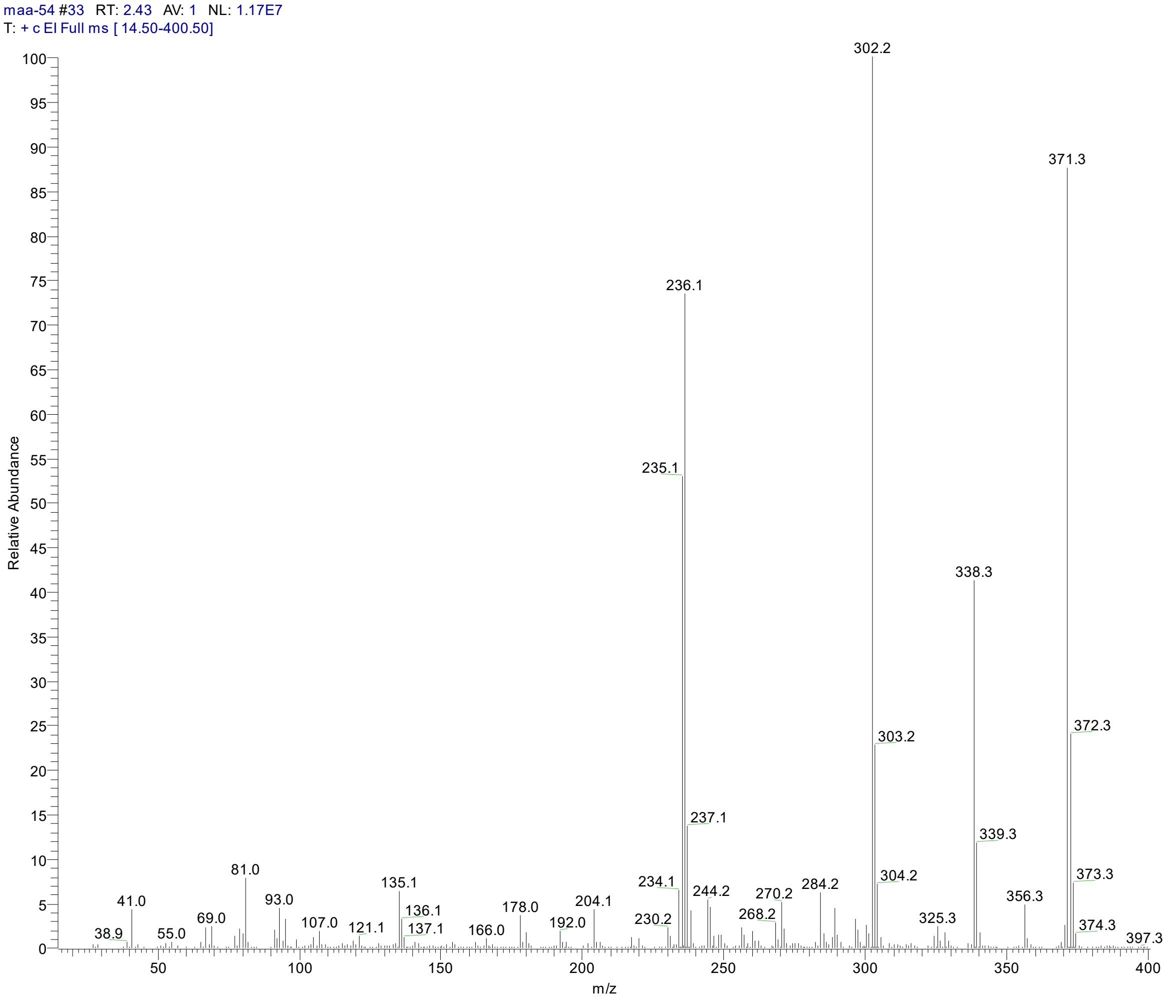

Figure S17: ^{1}H NMR of compound **20e**

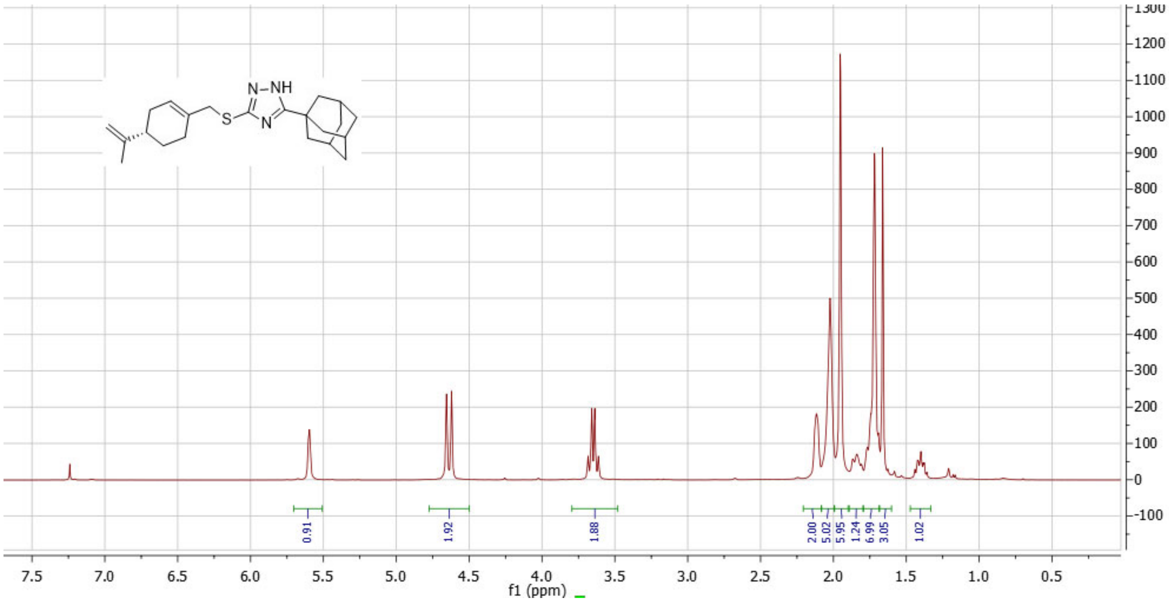

Figure S18: ^{13}C NMR of compound **20e**

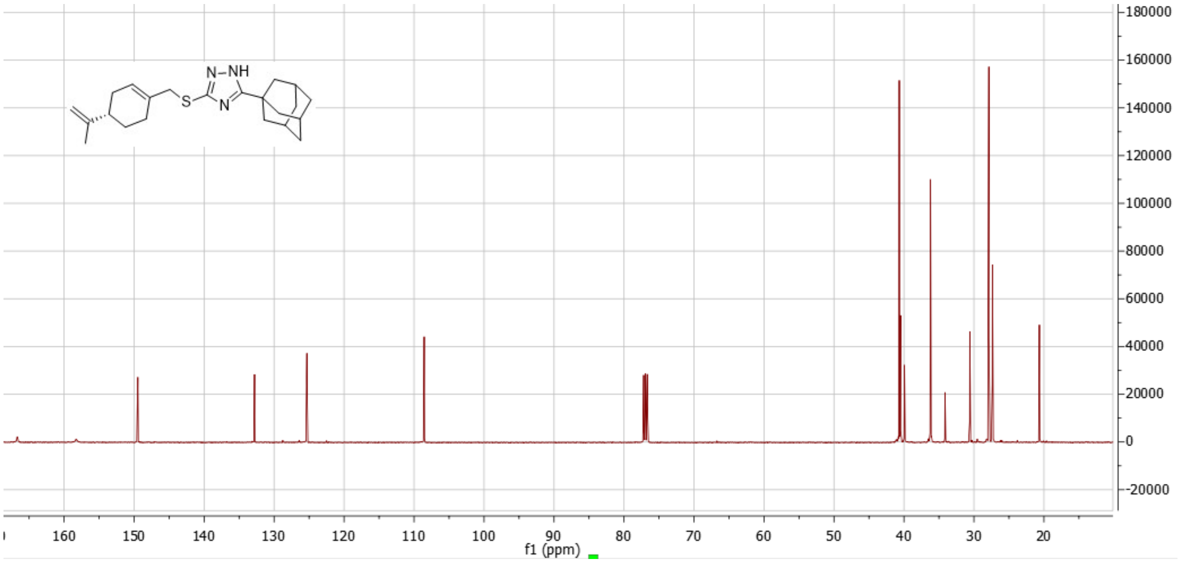

Figure S19: DFS spectrum of compound **20e**

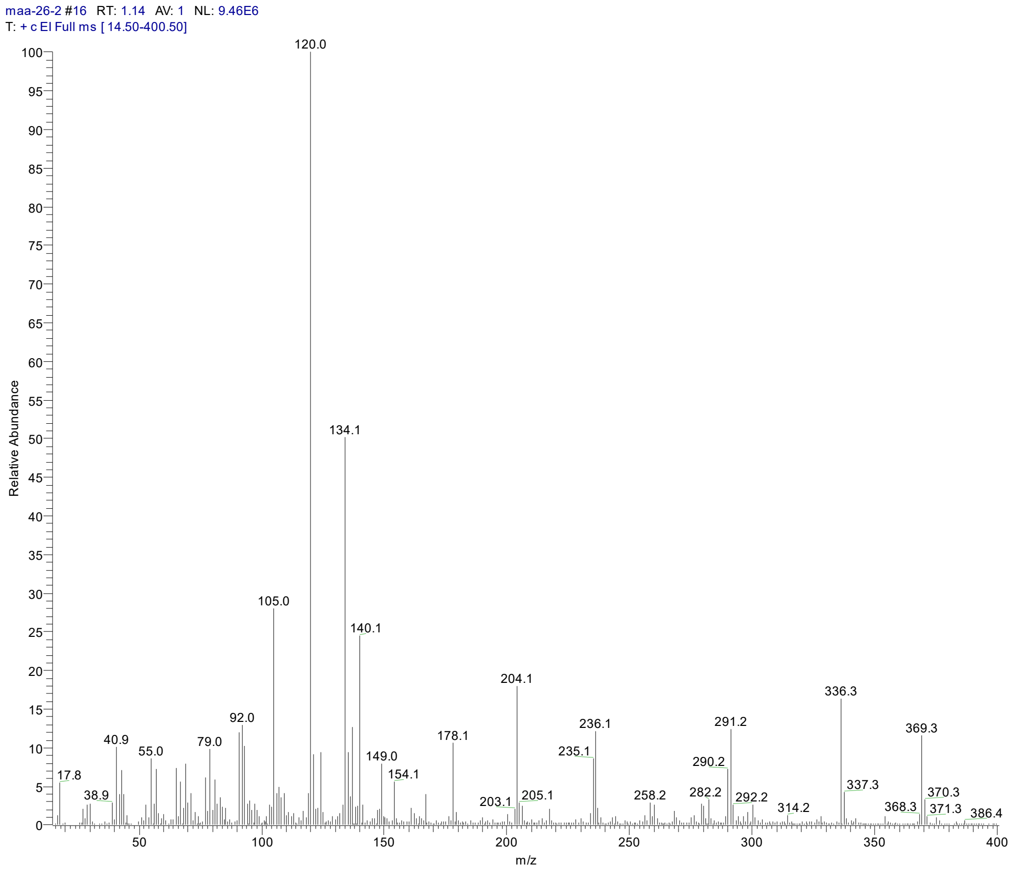

Figure S20: ^{1}H NMR of compound **20f**

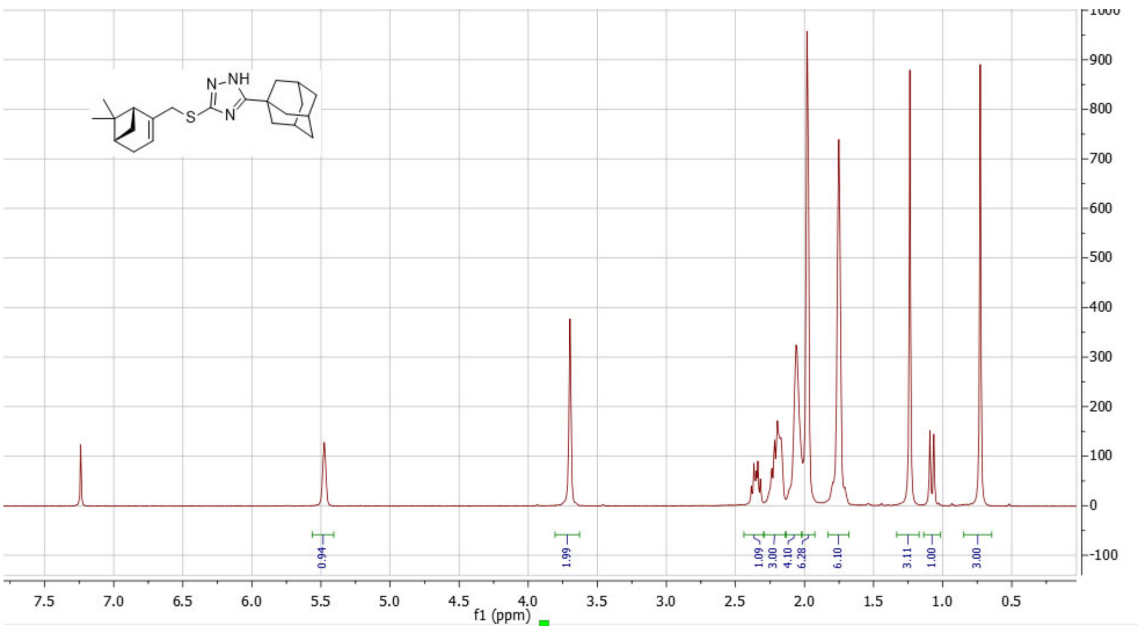

Figure S21: ^{13}C NMR of compound **20f**

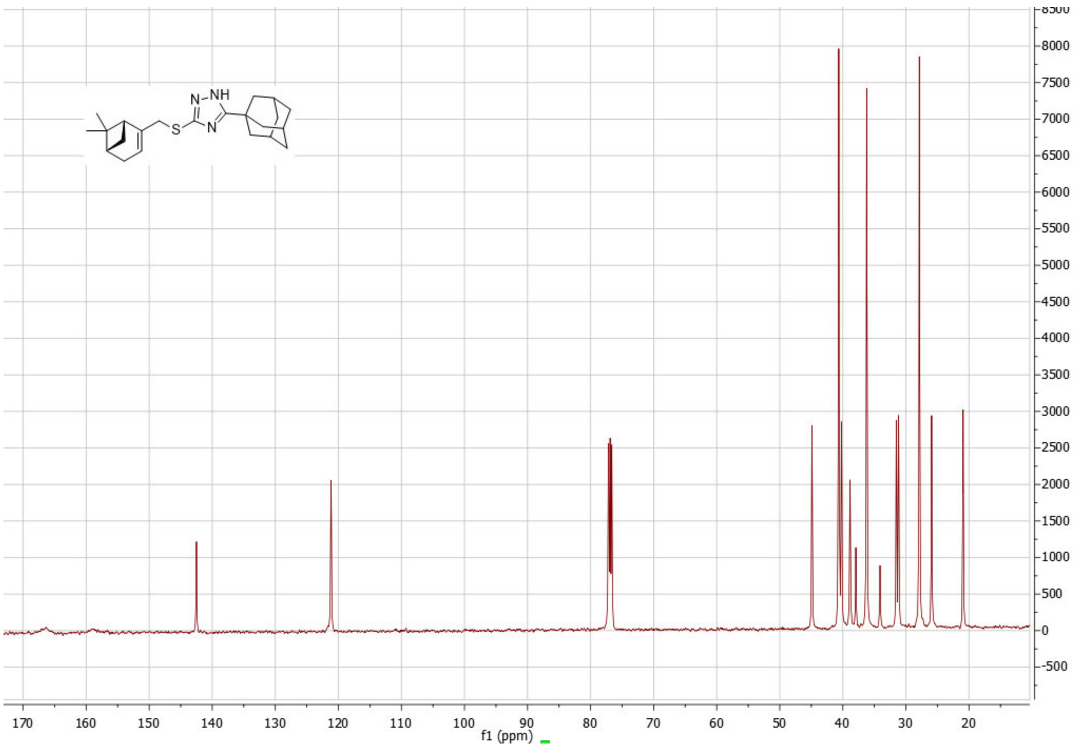

Figure S22: DFS spectrum of compound **20f**

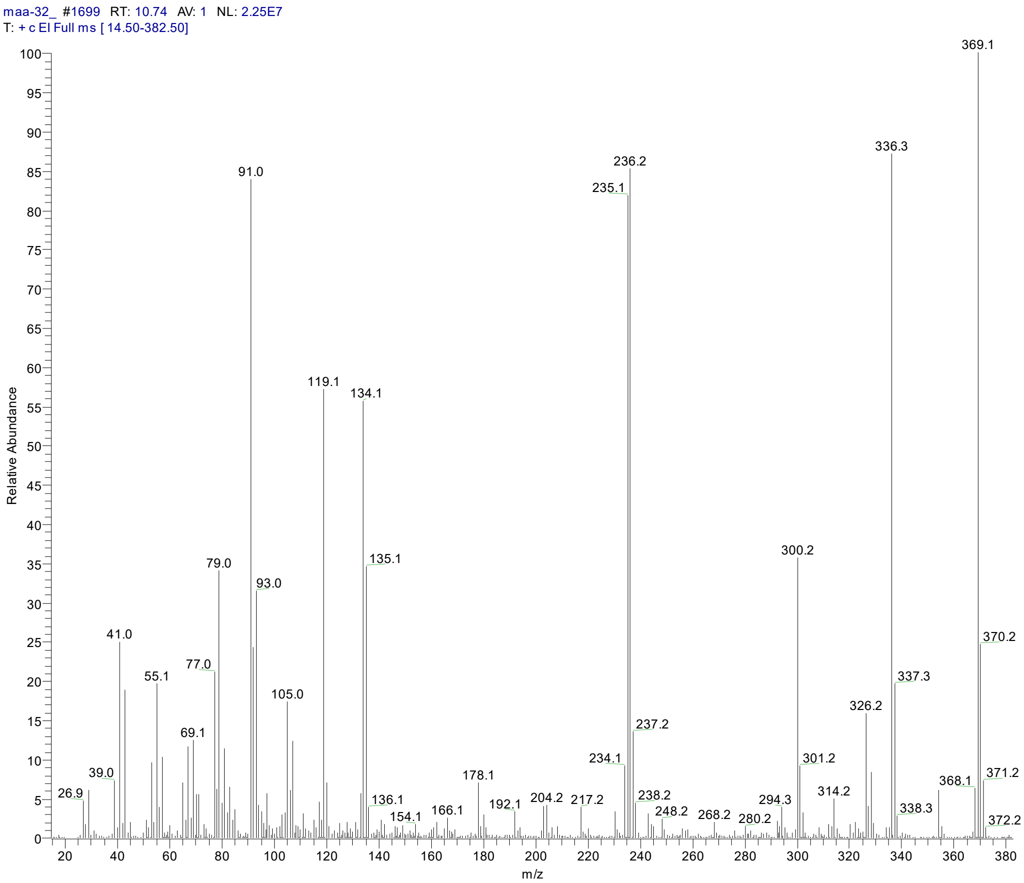

Figure S23: ^{1}H NMR of compound **20g**

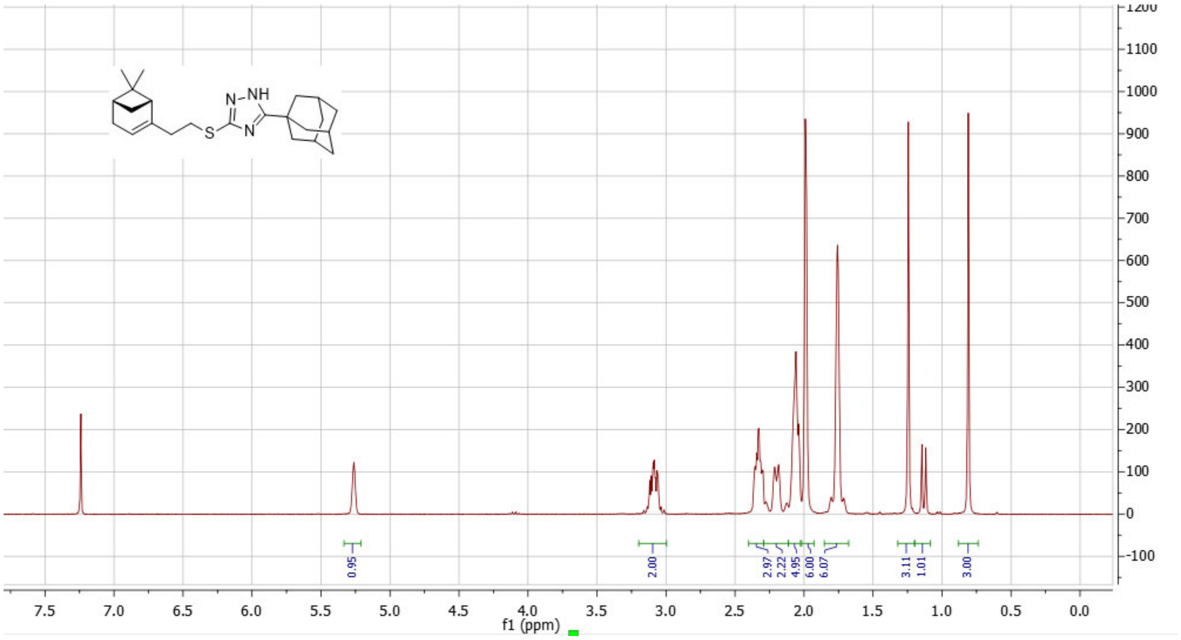

Figure S24: ^{13}C NMR of compound **20g**

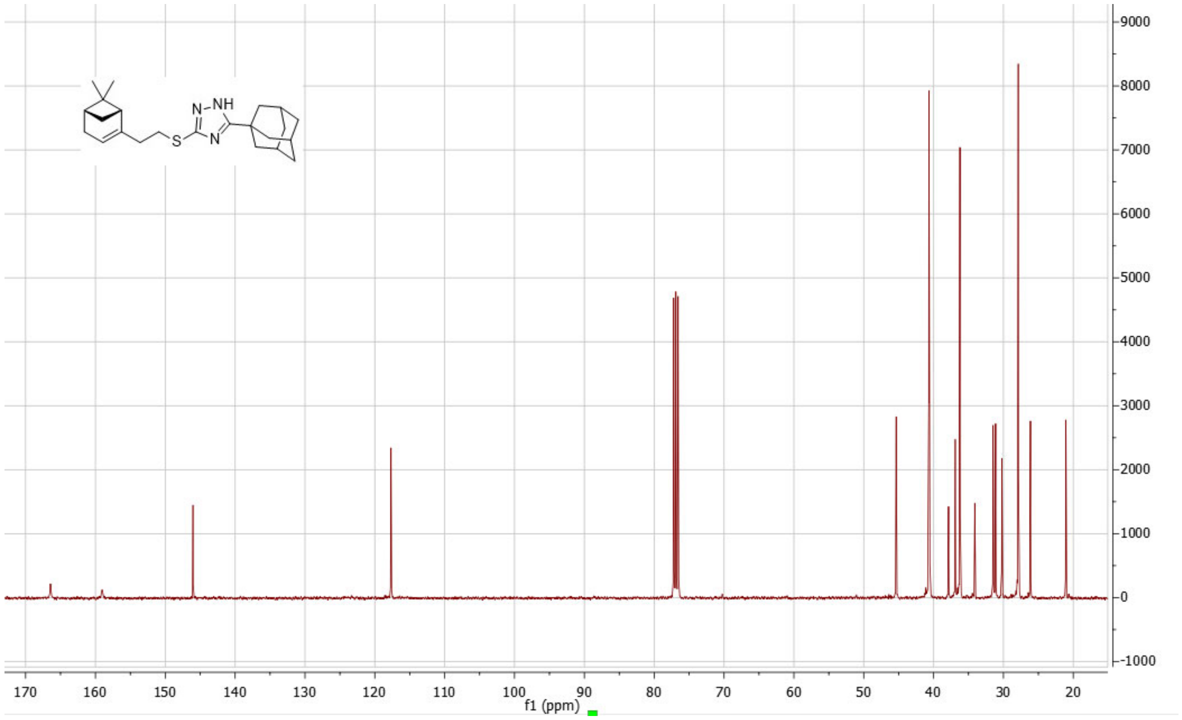

Figure S25: DFS spectrum of compound **20g**

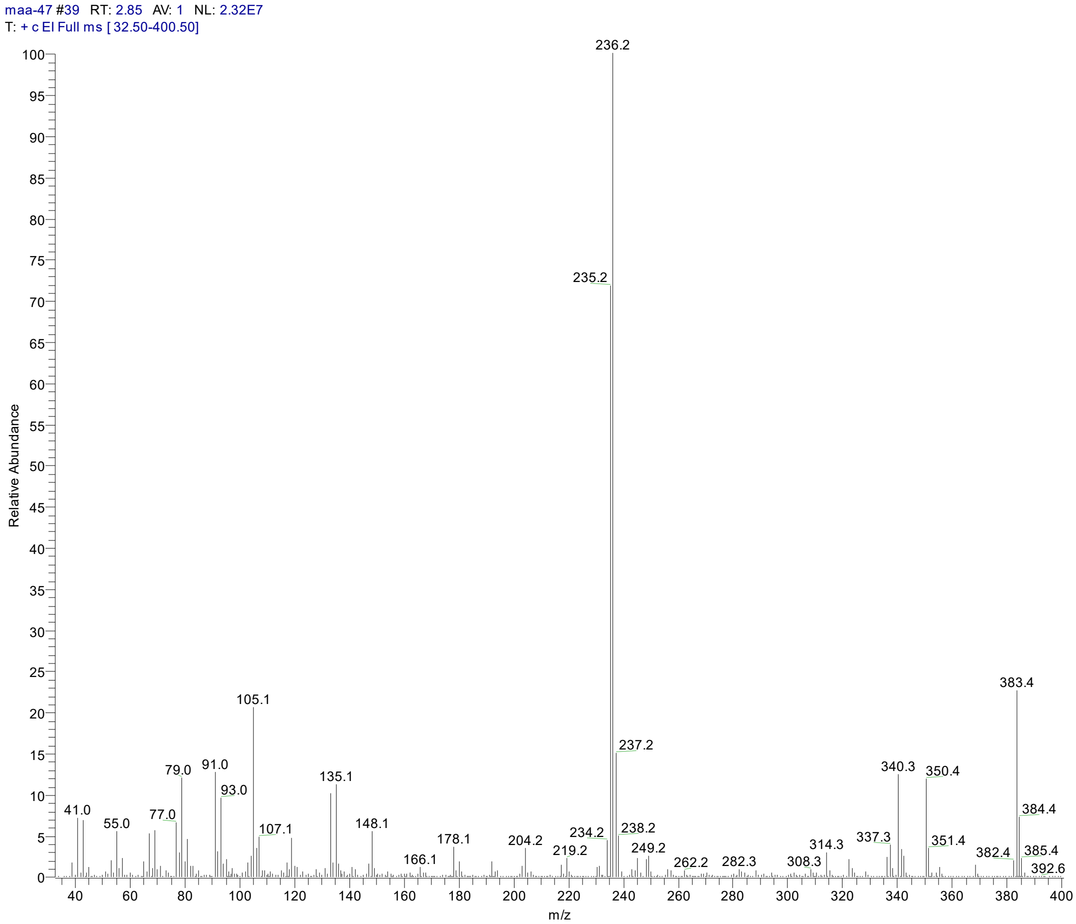

Figure S26: ^{1}H NMR of compound **25a**

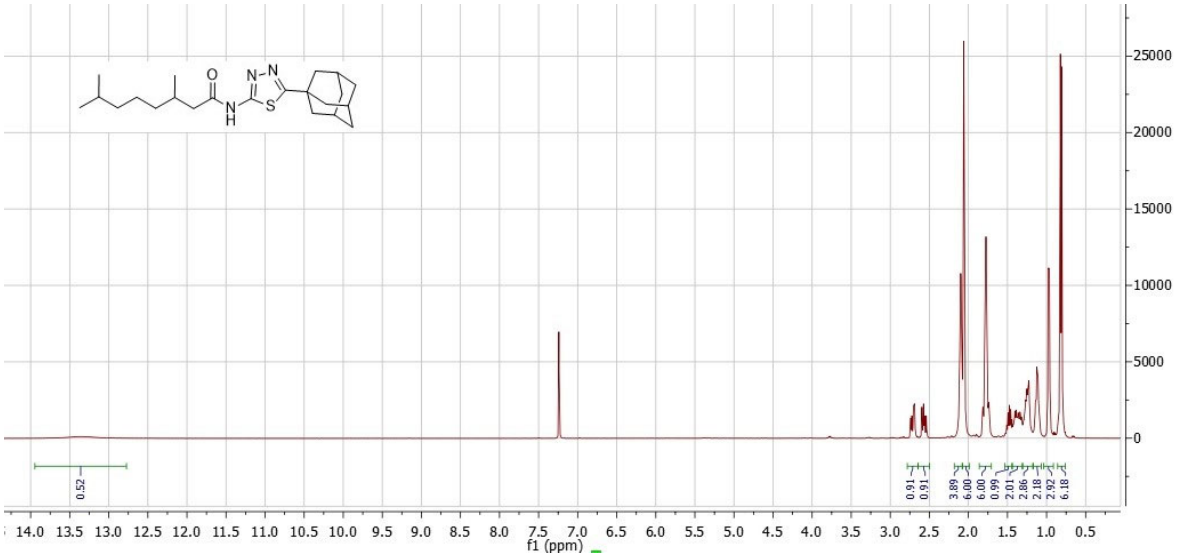

Figure S27: ^{13}C NMR of compound **25a**

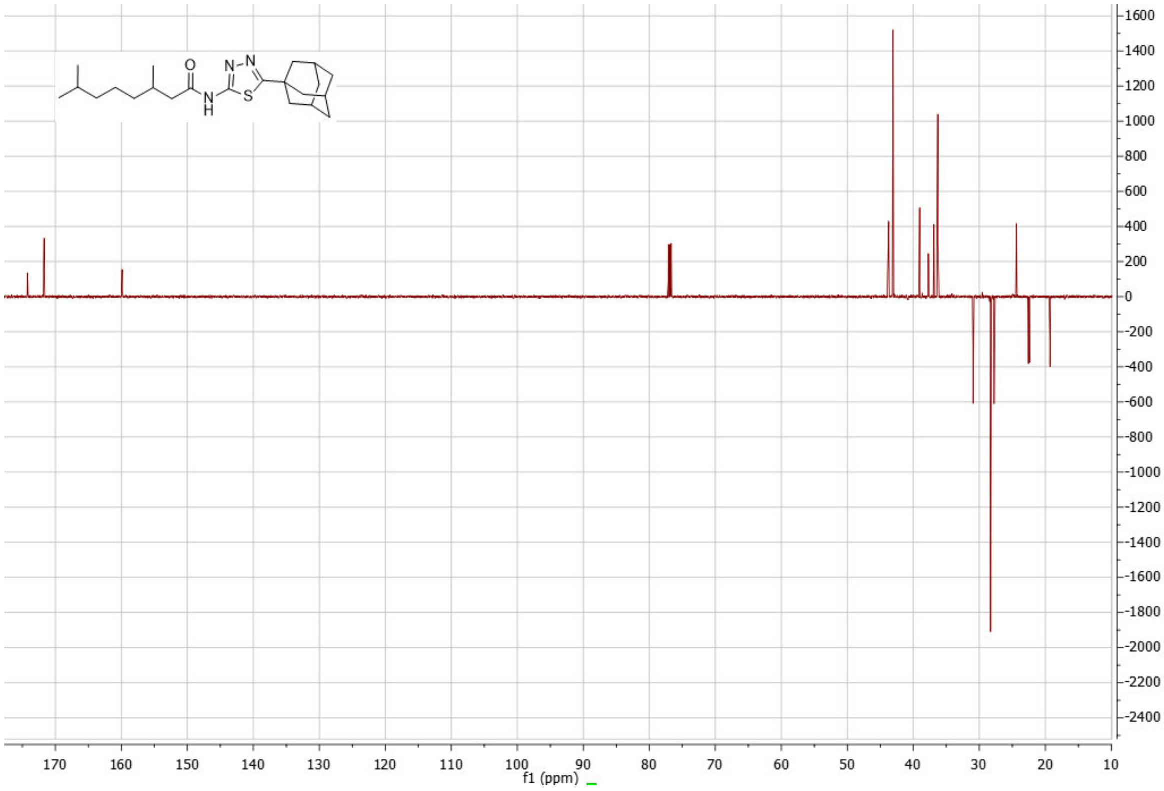

Figure S28: DFS spectrum of compound **25a**

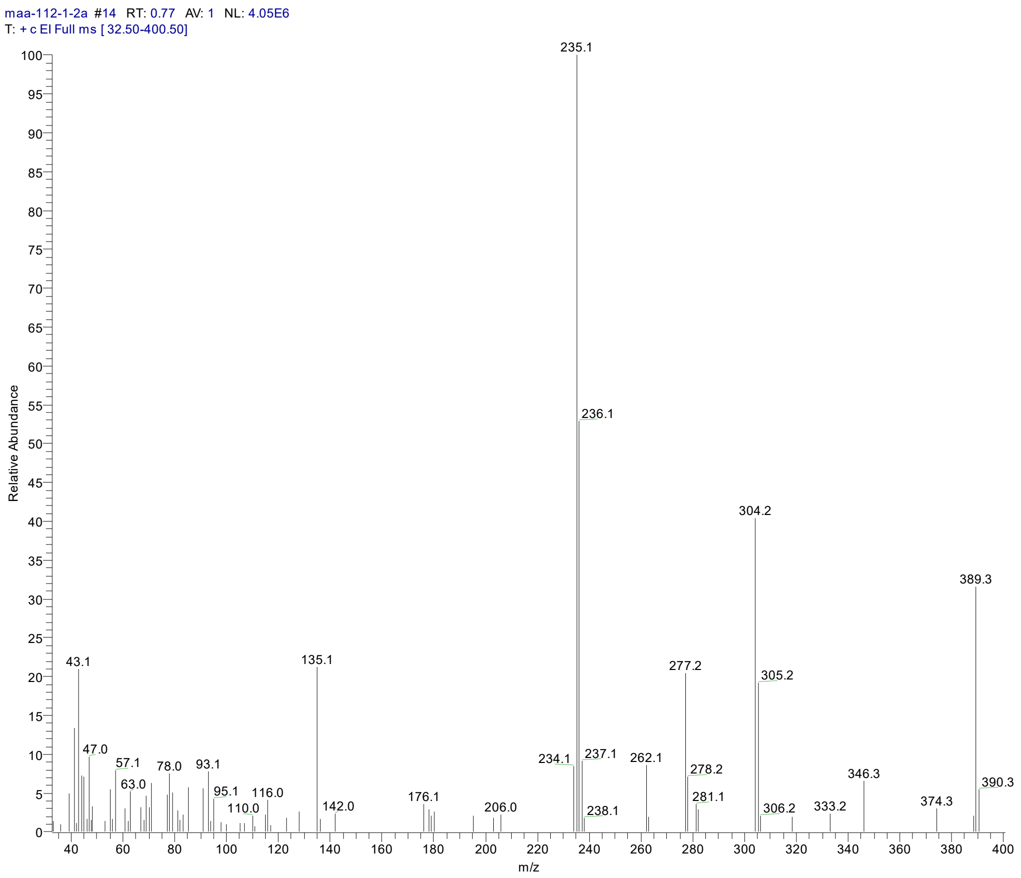

Figure S29: ^{1}H NMR of compound **25b**

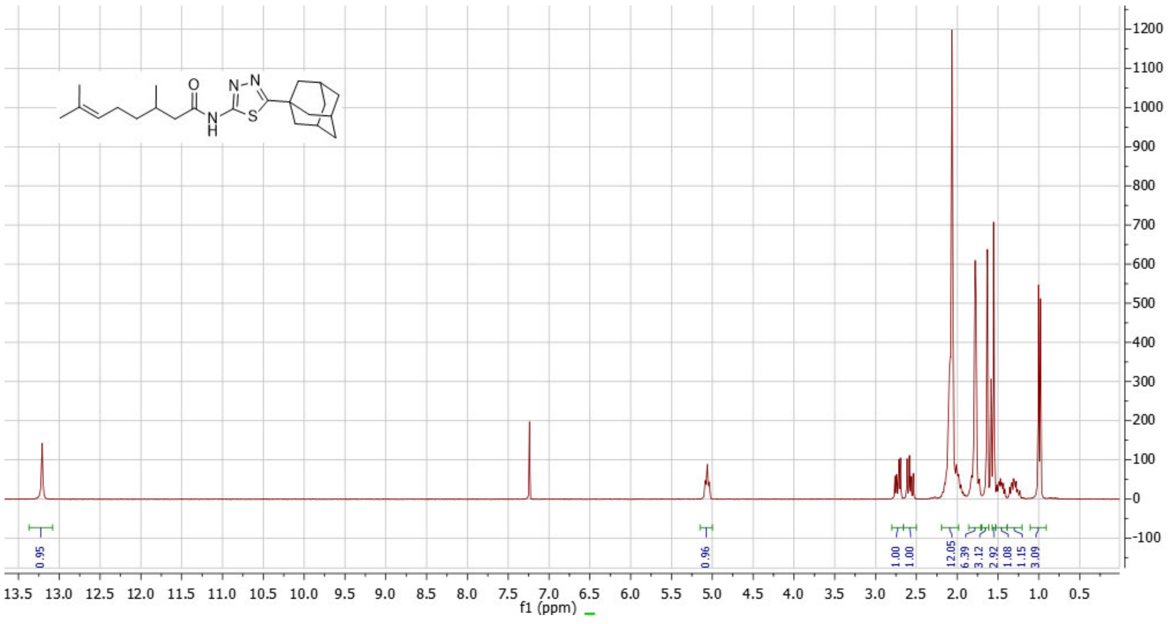

Figure S30: ^{13}C NMR of compound **25b**

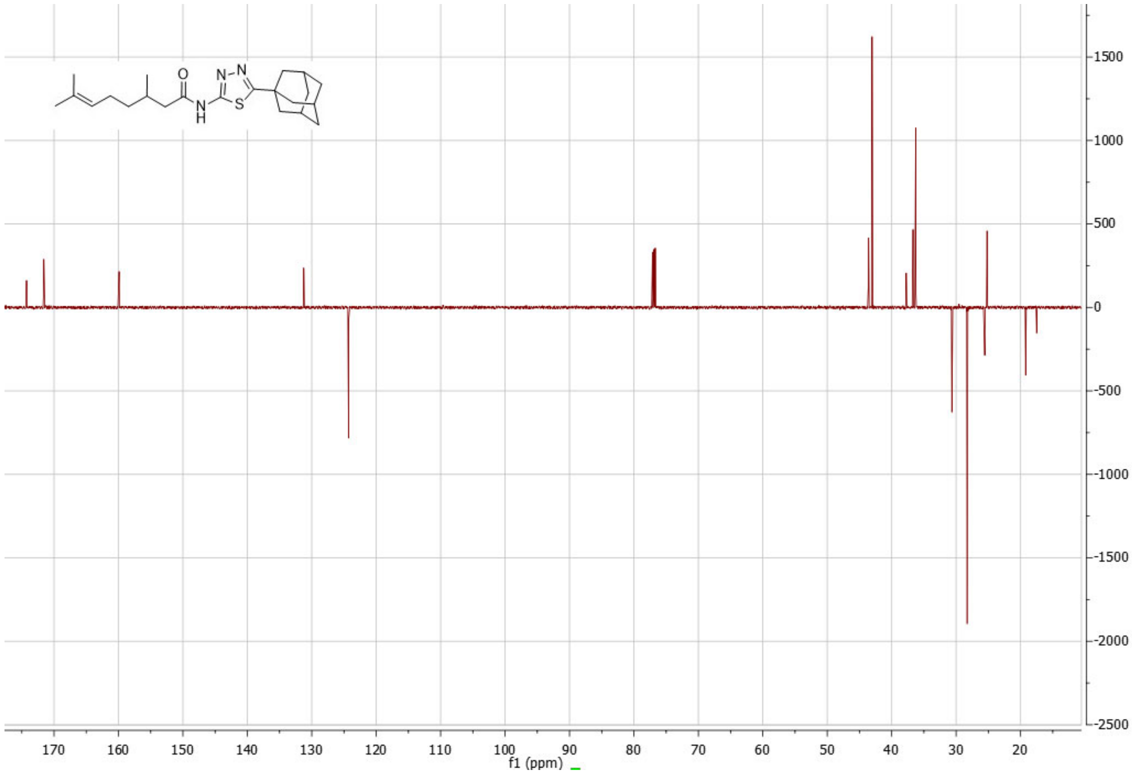

Figure S31: DFS spectrum of compound **25b**

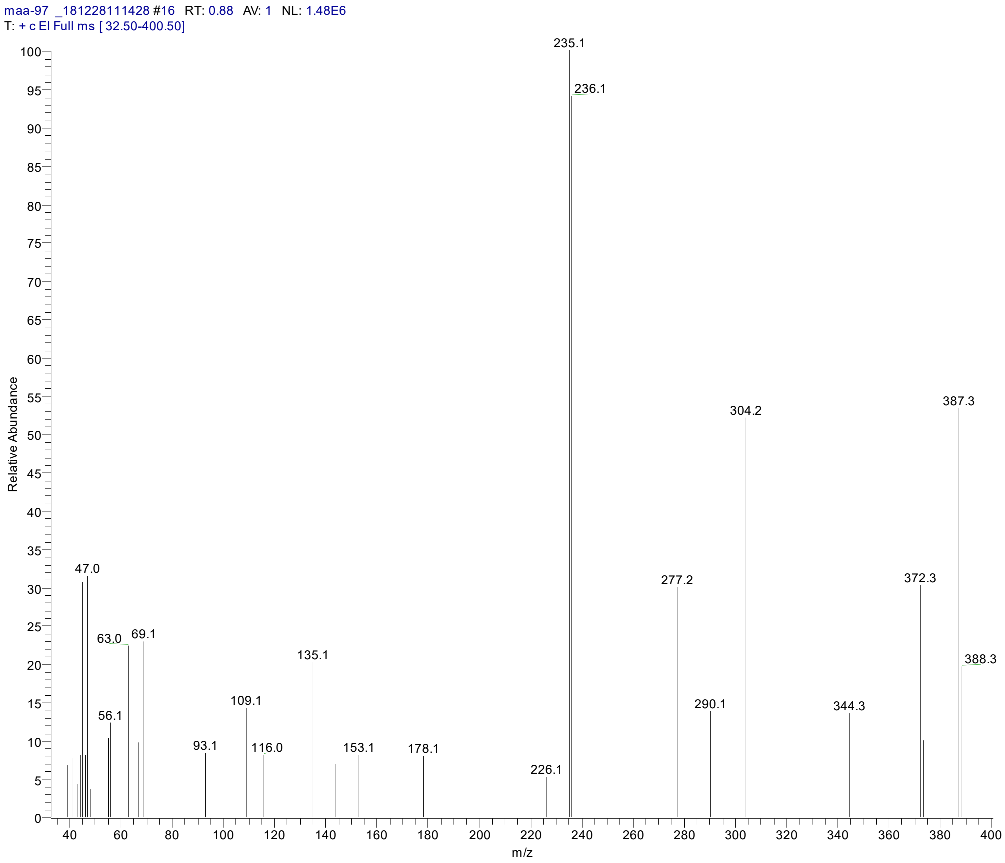

Figure S32: ^{1}H NMR of compound **25c**

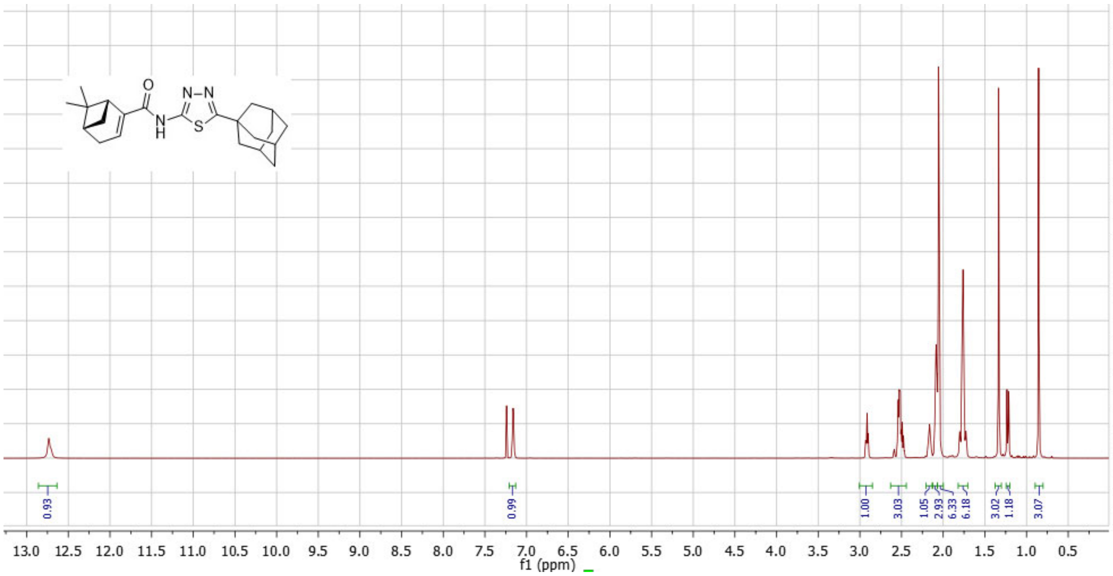

Figure S33: ^{13}C NMR of compound **25c**

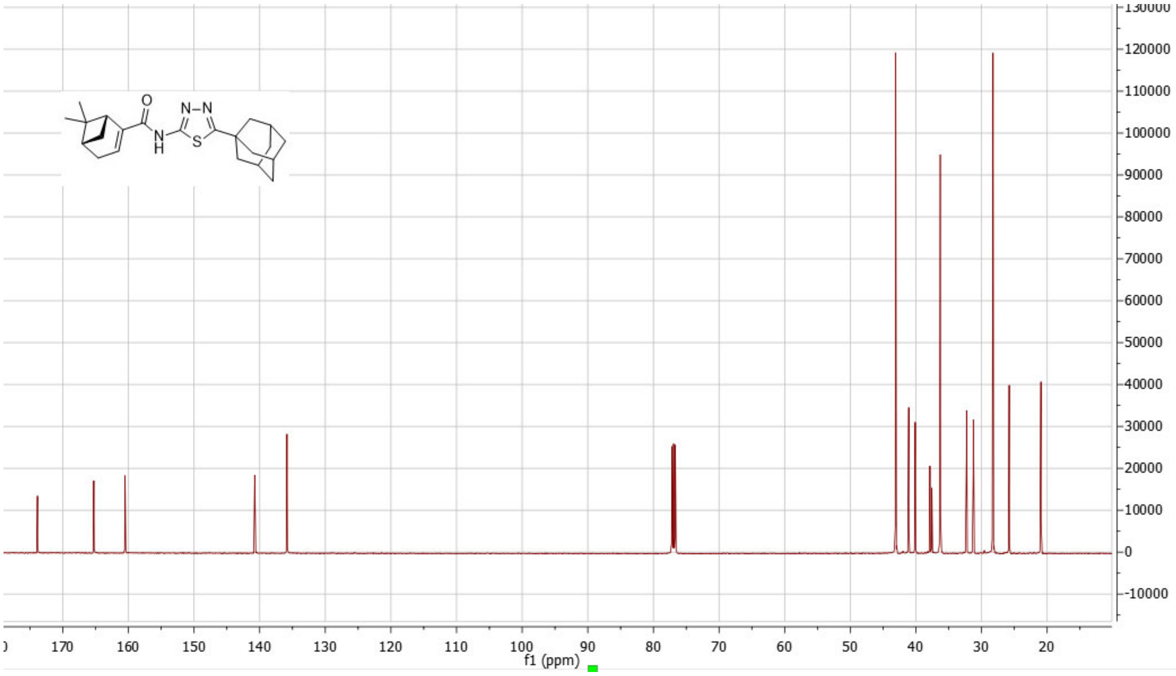

Figure S34: DFS spectrum of compound **25c**

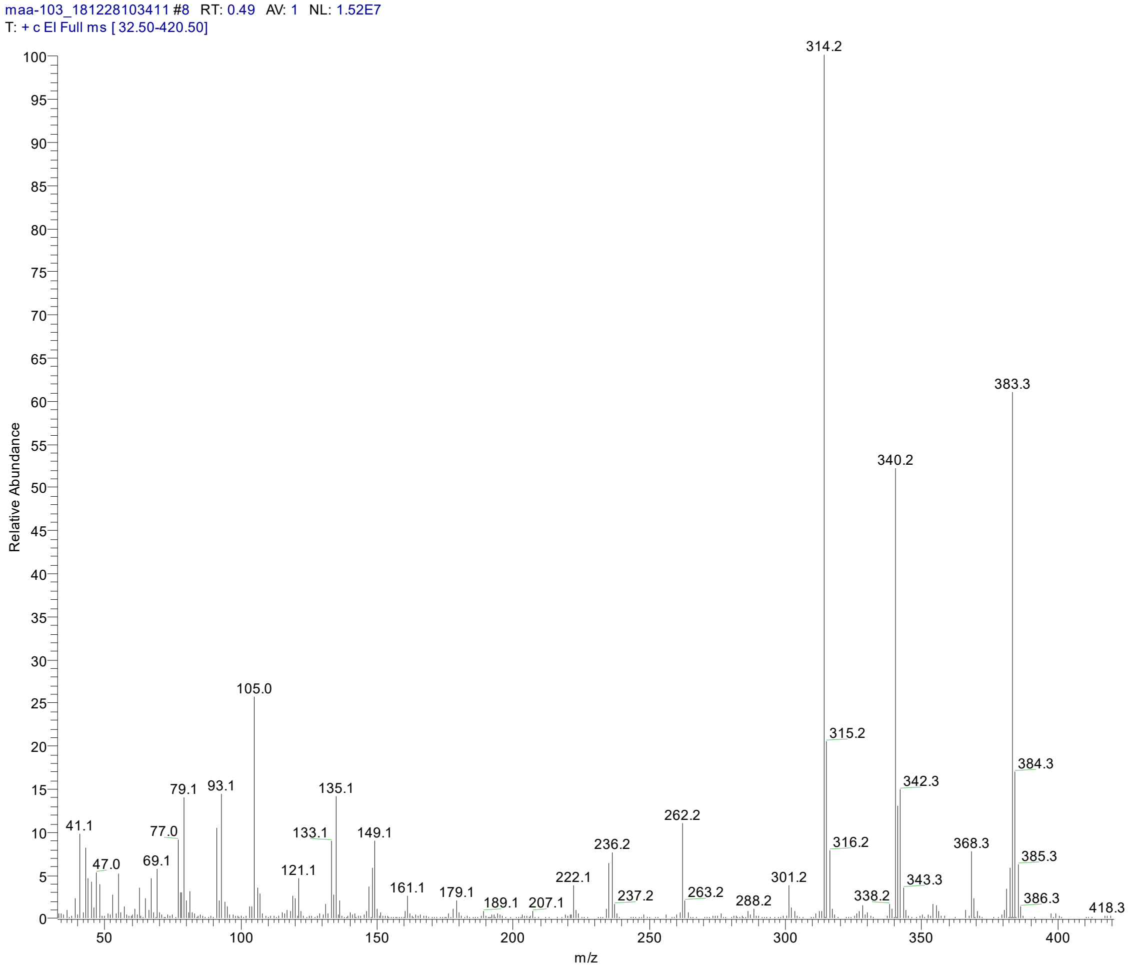

Figure S35: ^{1}H NMR of compound **25d**

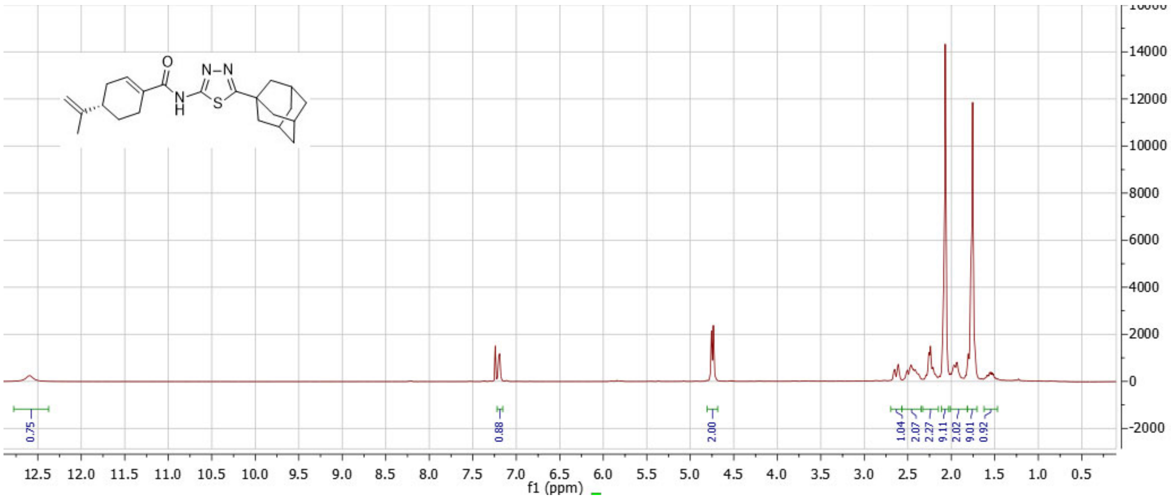

Figure S36: ^{13}C NMR of compound **25d**

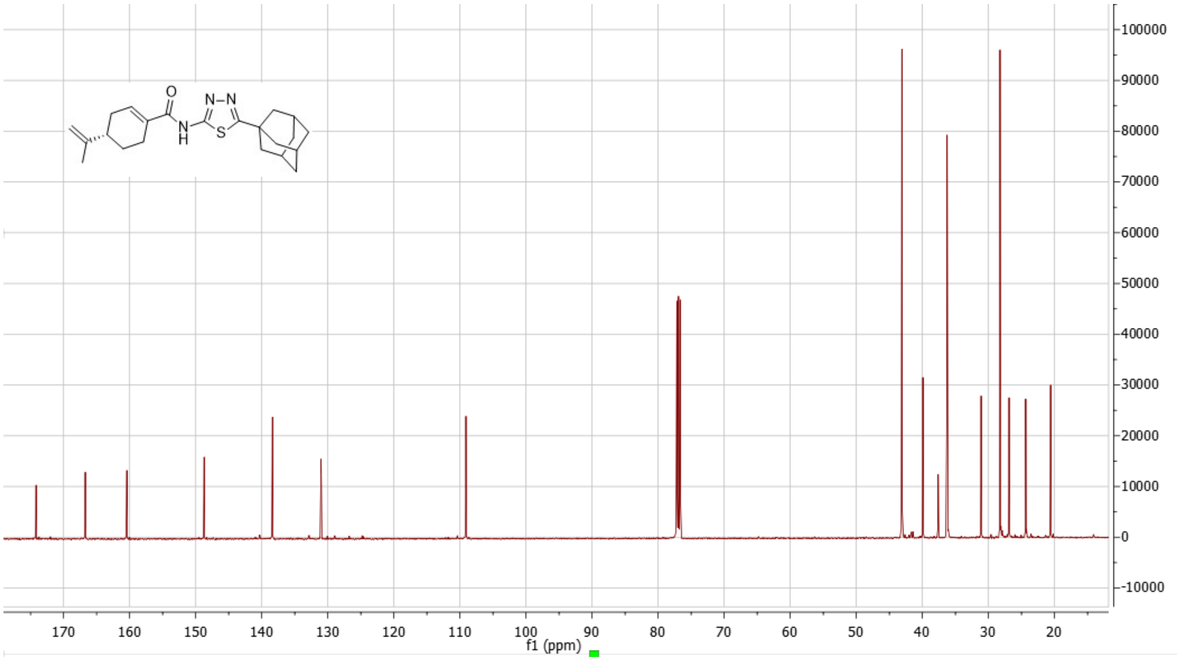

Figure S37: DFS spectrum of compound **25d**

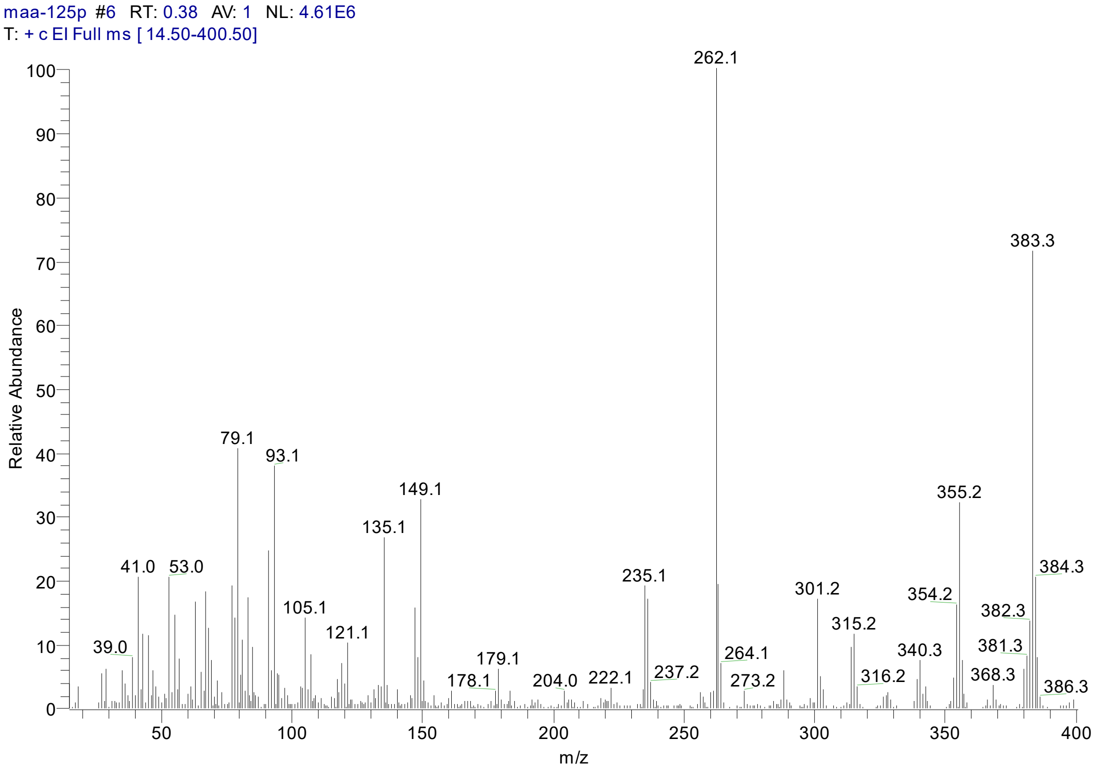

Figure S38: ^{1}H NMR of compound **26a**

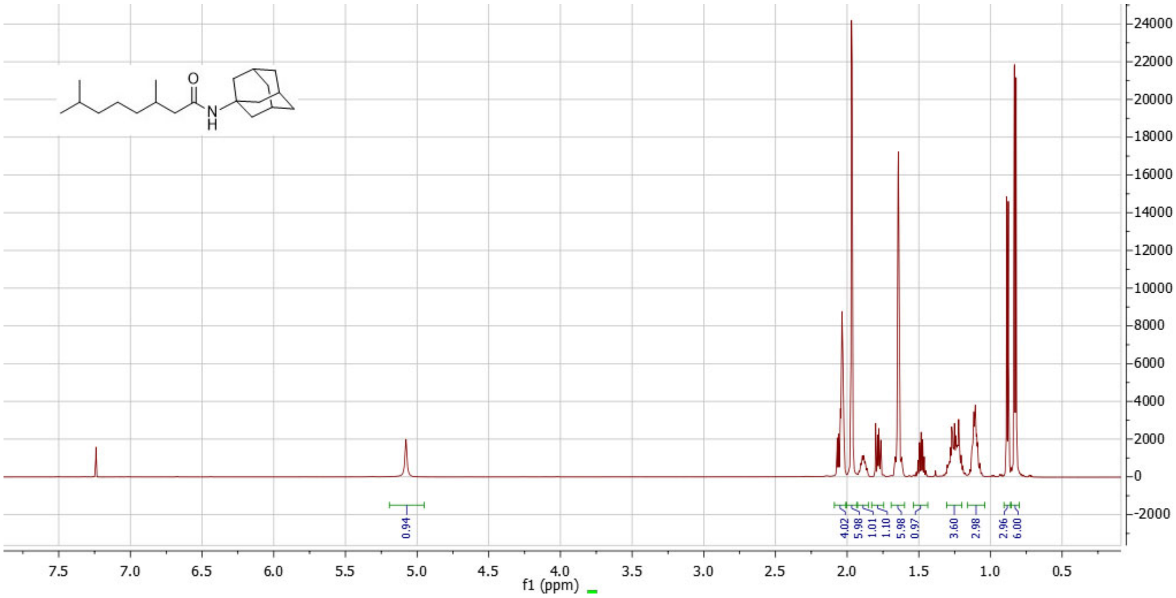

Figure S39: ^{13}C NMR of compound **26a**

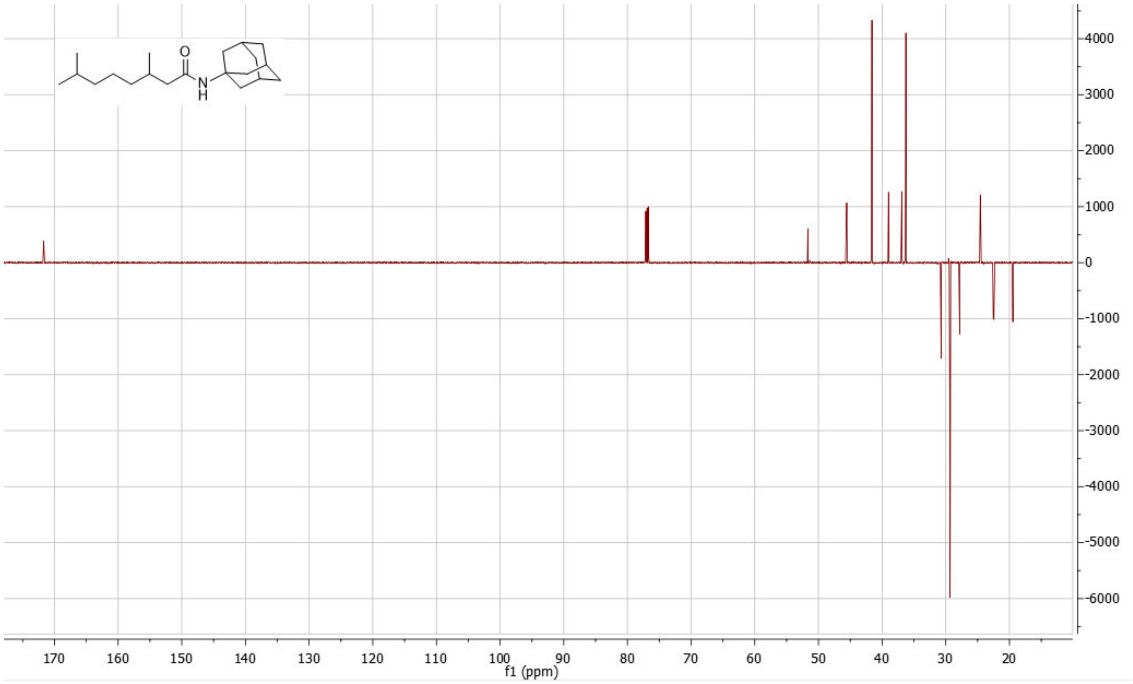

Figure S40: DFS spectrum of compound **26a**

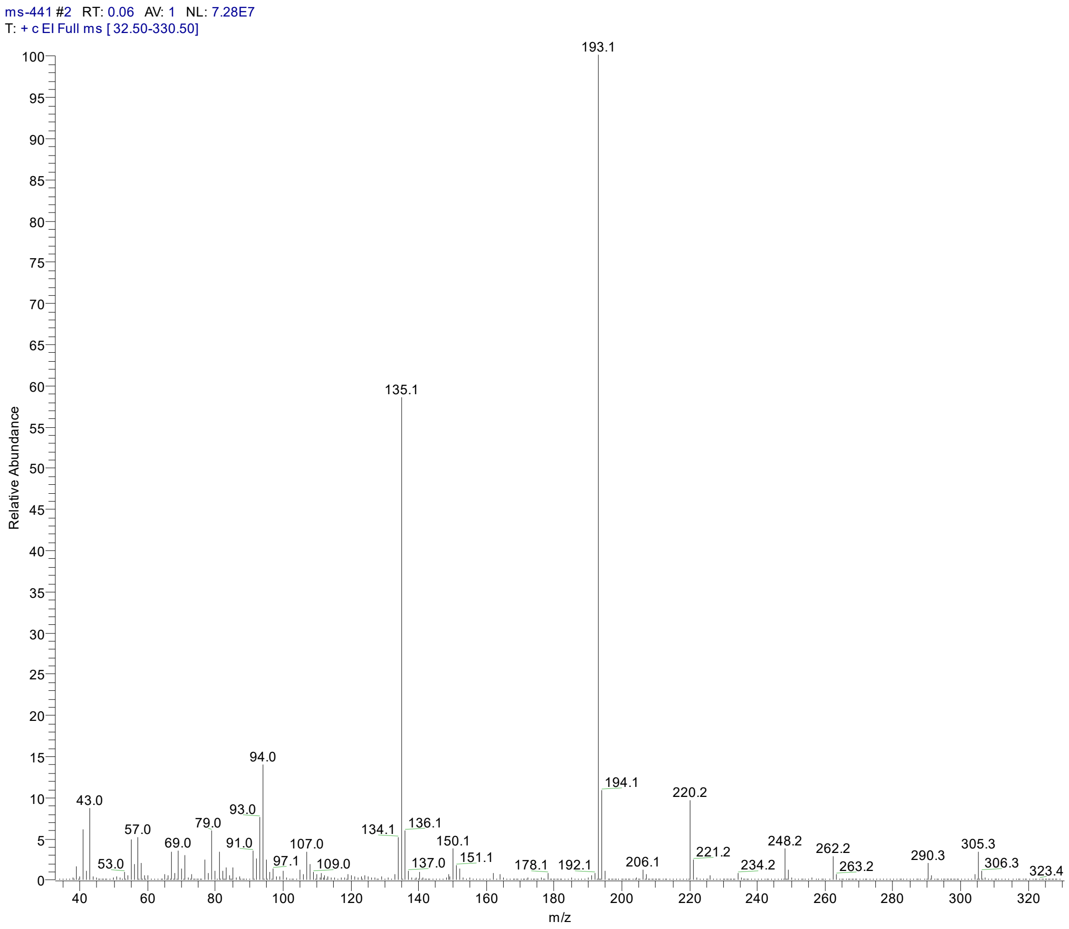

Table S1. The binding affinities as predicted by the scoring functions used.

Ligands	ASP	CS	ChemPLP	GS	IC_{50} (µM)
**20a**	32.8	27.4	60.5	69.0	0.54
**20b**	31.9	27.8	60.9	64.7	1.5
**20c**	32.1	27.9	62.6	70.2	5.3
**20d**	31.7	26.3	62.3	60.0	5.6
**20e**	32.3	27.6	61.0	68.0	6.2
**20f**	30.3	26.4	56.5	56.0	7.5
**20g**	31.3	27.8	58.6	55.8	0.57
**25a**	30.5	29.1	64.2	52.0	0.35
**25b**	28.5	27.7	63.0	54.4	2.59
**25c**	27.6	25.9	53.1	52.1	0.45
**28a**	27.2	27.5	57.4	49.1	>15
**28g**	28.2	28.1	61.6	51.3	>15
**26c**	22.8	25.7	47.5	43.3	>15
**Av. Act**	30.9±1.6	27.4±0.9	60.3±3.2	60.2±6.8	
**Av. >15**	26.1±2.3	27.1±1.0	55.5±5.9	47.9±3.4	

Table S2: The molecular descriptors and their corresponding Known Drug Indexes 2a and 2b ($KDI_{2a/2b}$). The R^2 numbers derived do not contain the IC_{50} > 15 µM values.

Ligands	RB	MW	HD	HA	Log P	PSA	KDI_{2A}	KDI_{2B}	IC_{50}, µM
**20a**	8	375.6	1	2.5	6.6	41.8	4.82	0.23	0.54
**20b**	7	373.6	1	2.5	6.5	42.1	4.93	0.27	1.5
**20c**	6	371.6	1	2.5	6.4	42.0	5.02	0.30	5.3
**20f**	3	369.6	1	2.5	5.8	42.0	5.15	0.37	7.5
**20g**	4	383.6	1	2.5	6.1	41.5	5.11	0.34	0.57
**25c**	2	383.6	1	4.5	4.7	62.6	5.59	0.64	0.45
**20d**	6	371.6	1	2.5	6.4	41.4	5.02	0.30	5.6
**20e**	4	369.6	1	2.5	6.0	42.1	5.15	0.36	6.2
**28a**	8	319.5	1	2.5	5.1	34.2	4.99	0.32	>15
**28g**	4	327.5	1	2.5	4.7	33.4	5.29	0.46	>15
**26c**	2	299.5	1	2.5	4.8	31.9	5.10	0.37	>15
**25a**	7	389.6	1	4.5	5.5	62.0	5.44	0.53	0.35
**25b**	6	387.6	1	4.5	5.4	61.8	5.53	0.59	2.59
**R^2**	0.0758	0.5638	0.000	0.2267	0.054	0.2242	0.0498	0.0819	

Table S3: Definition of lead-like, drug-like and Known Drug Space (KDS) in terms of molecular descriptors. The values given are the maxima for each descriptor for the volumes of chemical space used.

	**Lead-like Space**	**Drug-like Space**	**Known Drug Space**
Molecular weight (g mol^{-1})	300	500	800
Lipophilicity (Log P)	3	5	6.5
Hydrogen bond donors (HD)	3	5	7
Hydrogen bond acceptors (HA)	3	10	15
Polar surface area ($Å^2$) (PSA)	60	140	180
Rotatable bonds (RB)	3	10	17
